# Neuronally differentiated macula densa cells regulate tissue remodeling and regeneration in the kidney

**DOI:** 10.1172/JCI174558

**Published:** 2024-04-10

**Authors:** Georgina Gyarmati, Urvi Nikhil Shroff, Anne Riquier-Brison, Dorinne Desposito, Wenjun Ju, Sean D. Stocker, Audrey Izuhara, Sachin Deepak, Alejandra Becerra Calderon, James L. Burford, Hiroyuki Kadoya, Ju-Young Moon, Yibu Chen, Markus M. Rinschen, Nariman Ahmadi, Lester Lau, Daniel Biemesderfer, Aaron W. James, Liliana Minichiello, Berislav V. Zlokovic, Inderbir S. Gill, Matthias Kretzler, János Peti-Peterdi

**Affiliations:** 1 Department of Physiology and Neuroscience and Department of Medicine, Zilkha Neurogenetic Institute, University of Southern California, Los Angeles, California, USA.; 2 Division of Nephrology, Department of Medicine, and Department of Computational Medicine and Bioinformatics, University of Michigan, Ann Arbor, Michigan, USA.; 3 Department of Neurobiology, University of Pittsburgh School of Medicine, Pittsburgh, Pennsylvania, USA.; 4 USC Libraries Bioinformatics Service, University of Southern California, Los Angeles, California, USA.; 5 Center for Molecular Medicine, Faculty of Medicine and University Hospital Cologne, University of Cologne, Cologne, Germany.; 6 Institute of Urology, Catherine and Joseph Aresty Department of Urology, University of Southern California, Los Angeles, California, USA.; 7 Department of Biochemistry and Molecular Genetics, College of Medicine, The University of Illinois at Chicago, Chicago, Illinois, USA.; 8 Section of Nephrology and Department of Internal Medicine, Yale University School of Medicine, New Haven, Connecticut, USA.; 9 Department of Pathology, Johns Hopkins University, Baltimore, Maryland, USA.; 10 Department of Pharmacology, University of Oxford, Oxford, United Kingdom.

**Keywords:** Nephrology, Chronic kidney disease

## Abstract

Tissue regeneration is limited in several organs, including the kidney, contributing to the high prevalence of kidney disease globally. However, evolutionary and physiological adaptive responses and the presence of renal progenitor cells suggest an existing remodeling capacity. This study uncovered endogenous tissue remodeling mechanisms in the kidney that were activated by the loss of body fluid and salt and regulated by a unique niche of a minority renal cell type called the macula densa (MD). Here, we identified neuronal differentiation features of MD cells that sense the local and systemic environment and secrete angiogenic, growth, and extracellular matrix remodeling factors, cytokines and chemokines, and control resident progenitor cells. Serial intravital imaging, MD nerve growth factor receptor and Wnt mouse models, and transcriptome analysis revealed cellular and molecular mechanisms of these MD functions. Human and therapeutic translation studies illustrated the clinical potential of MD factors, including CCN1, as a urinary biomarker and therapeutic target in chronic kidney disease. The concept that a neuronally differentiated key sensory and regulatory cell type responding to organ-specific physiological inputs controls local progenitors to remodel or repair tissues may be applicable to other organs and diverse tissue-regenerative therapeutic strategies.

## Abstract 2

Physiological-based activation of the key sensory and regulatory renal cell type in the macula densa regulates resident progenitor cells and secretes multiple tissue remodeling factors.

## Graphical abstract

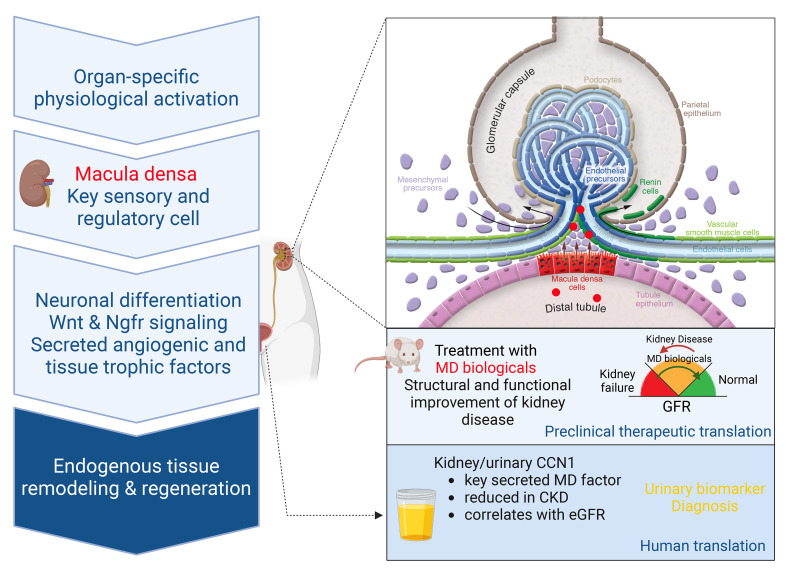

## Introduction

Regeneration of most tissues and organs in mammalians, particularly in humans, is limited (1), and the kidney is a prime example (2). Despite recent advances in regrowing or repairing kidney tissues, chronic kidney disease (CKD) remains a major global health issue, and its incidence continues to rise (2). The classic and current standard-of-care therapies are nonspecific and can only slow down CKD progression to end-stage renal disease (3, 4), resulting in the urgent and unmet medical need for highly efficient CKD therapies for the treatment of millions of patients worldwide. Improved understanding of endogenous kidney tissue repair and identification of the key cellular and molecular targets are critically important for the development of specific, mechanism-based regenerative therapies that can achieve disease regression.

Physiological adaptation to maintain functional homeostasis is a known driver of physiological regeneration in multiple organisms and mammalian tissues (1). Physiological signals associated with either loss or gain of organ function, such as fasting for the nervous, endocrine, and digestive systems (5, 6) and mechanical force for the skeletal system (7), can trigger potent regenerative responses. Additional clues regarding endogenous regenerative mechanisms come from adaptive evolution. Conservation of body fluid and salt was a major driver of evolution that brought about the highly efficient and complex mammalian nephron compared with the primitive structure in fish (8). The loss of neonephrogenesis capacity in birds and mammals coincided with the appearance of a cell-based regeneration strategy and the differentiation of unique, specialized nephron cell types (8), including the macula densa (MD). The MD is a main salt sensor in each nephron that is formed by a niche of about 25 individual MD cells localized in a strategically central position at the vascular pole entrance of the kidney filter (glomerulus). MD cells via salt sensing are known to provide key physiological control of basic kidney functions, including glomerular filtration rate, renal blood flow, and renin release (9, 10), but the true nature of these cells has been unknown due to their inaccessibility. According to the existing paradigm, MD cells can sense alterations in renal tubular fluid variables, including NaCl content and metabolic intermediates, that via various intracellular signaling pathways trigger the synthesis and release of chemical mediators. These mediators then in turn act in a paracrine fashion on final effector cells, including renin-producing cells, to secrete renin or contractile vascular smooth muscle cells of glomerular arterioles to regulate vascular resistance and organ blood flow via tubuloglomerular feedback (TGF) (9, 10). The MD-specific expression of the neuronal type of nitric oxide synthase (NOS1) in the renal cortex has been long established (11). The recent microanatomical discovery of long, axon-like MD cell processes (12) and preliminary transcriptomic analysis of a limited number of single MD cells (13) suggest their neuronal differentiation.

Recent discoveries in tissue regenerative mechanisms suggest the key role of local neuronal activity in direct control of somatic stem cells. Peripheral sensory and sympathetic nerves have been demonstrated to drive epidermal stem cells in wound repair (14) and hair follicle stem cells, including melanocytes (15, 16), respectively. The emerging key role of local neurons in endogenous tissue repair fits well with the clues on MD cell neuronal differentiation, sensing, and responding to the environment and with their potential links to physiological tissue regeneration.

Here, we addressed the hypotheses that neuronally differentiated MD cells are important regulators of mesenchymal and endothelial progenitor cells and endogenous kidney tissue remodeling and that they secrete multiple angiogenic, growth, and extracellular matrix remodeling factors and chemokines that can be targeted in regenerative therapies. We further hypothesized that these MD functions can be augmented by the loss of body fluid and salt, which is the primary, organ-specific, and evolution-driven physiological signal for the kidney. Consistent with physiological activation being the overarching theme of this work, the same low dietary salt intake stimulatory condition was applied throughout all parts of the study, including in vivo animal models, transcriptomic analysis, and in vitro cell culture, and, as the ultimate goal, for the human and therapeutic translation of the presently identified tissue remodeling mechanisms.

## Results

### Live serial tracking of endogenous tissue remodeling.

**Figure 1:**
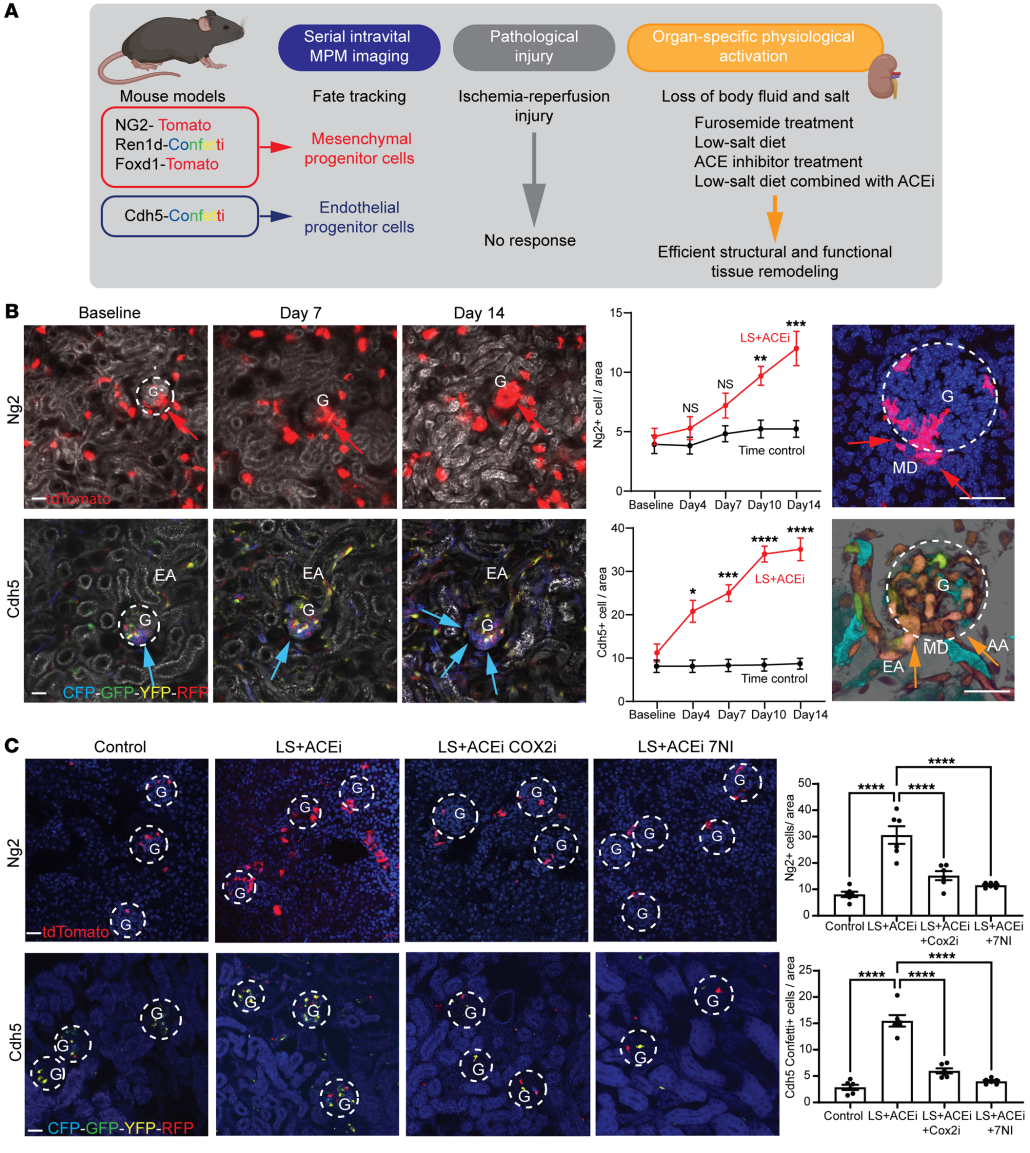
Evaluation of mesenchymal (Ng2-tdTomato, red) and endothelial (Cdh5-Confetti, multicolor) precursor cell–mediated endogenous kidney tissue remodeling. (**A**) Experimental design for study of endogenous tissue remodeling using serial multiphoton microscopy (MPM) combined with multiple genetic reporter mouse models in physiological and pathological conditions. (**B**) Representative in vivo MPM maximum projection images (left) and the number of Ng2^+^ or Cdh5^+^ cells per glomerular area (center) of the same kidney cortex area/volume visualized through an abdominal imaging window at the indicated time points or in a magnified single glomerulus (right, on day 14). Responses to low-salt (LS) diet and ACEi (all images) or timed control (center) are shown; *n* = 6 (1–2 glomeruli/animal). Note the low cell number and random distribution at baseline but high cell number specifically at the glomerular vascular pole (red and blue arrows) and the Cdh5^+^ clones (CFP/blue, left) and YFP/RFP (orange, right) among all 10 different CFP/GFP/YFP/RFP Confetti color combinations. Plasma was labeled with Alexa Fluor 594–albumin (gray scale). The *Z*-stacks of same preparations are shown in Supplemental Video 1. G, glomerulus (dashed white circles); AA/EA, afferent/efferent arteriole; MD, macula densa. (**C**) The effects of LS diet + ACEi for 10 days with or without selective COX2 inhibition with SC58236 or NOS1 inhibition with 7-NI treatment, or 10 days timed control on Ng2^+^ and Cdh5^+^ cell number per glomerular area analyzed on frozen tissue sections; *n* = 6 (average of 10 glomeruli/animal). Nuclei were labeled blue with DAPI. Scale bars: 50 μm. Data represent mean ± SEM. *P < 0.05, **P < 0.01, ***P < 0.001, ****P < 0.0001, ANOVA followed by Dunnett’s test.

To establish the dynamics and pattern of endogenous kidney tissue remodeling with single-cell resolution in the intact living mouse kidney, unbiased tracking of the same tissue volume of kidney cortex over several days and weeks was performed using a combination of serial intravital multiphoton microscopy (MPM) (17, 18) and genetic cell fate tracking. The applied experimental strategy, conditions, and inducible fluorescent reporter mouse models to genetically label and track various mesenchymal and endothelial cell lineages are summarized in Figure 1A. In contrast to the lack of effect of pathological injury, such as in the ischemia/reperfusion model of acute kidney injury (confirmed by KIM1^+^ labeling, Supplemental Figure 1A; supplemental material available online with this article; https://doi.org/10.1172/JCI174558DS1) or time control (Supplemental Figure 1B), the loss of body fluid and salt as a MD-activating physiological stimulus using treatment with low-salt (LS) diet and the angiotensin-converting enzyme inhibitor enalapril (ACEi) for up to 2 weeks caused substantial recruitment of mesenchymal and endothelial precursor cells in Ng2-tdTomato and Cdh5-Confetti mice (19), respectively (Figure 1B and Supplemental Video 1). Importantly, the geometrical epicenter (highest cell density) of mesenchymal and endothelial cell recruitment was near the base of MD cells at the glomerular vascular pole in each nephron (Figure 1B). Other physiology-based stimuli that are known to trigger MD cell sensing of LS (the diuretic furosemide, LS diet, or ACEi alone) caused similar positive but less robust responses, while high-salt diet had a negative effect (Supplemental Figure 1A). Newly recruited Ng2^+^ cells differentiated to multiple cell fates, including vascular smooth muscle, renin, mesangial, parietal epithelial, and proximal tubule cells and podocytes (Supplemental Figure 1C). Alternative genetic strategies to track mesenchymal cell lineages (Ren1d-Confetti and Foxd1-tdTomato mice) produced similar results (Supplemental Figure 1, D and E). Both Cdh5^+^ and Ren^+^ cells produced clonal remodeling of the vasculature, interstitium, and glomerulus closest to the MD (Figure 1B and Supplemental Figure 1, D and E), further suggesting the presence of mesenchymal and endothelial progenitor cells at the glomerular vascular pole. LS plus ACEi-induced Ng2^+^ and Cdh5^+^ cell recruitment to the glomerular vascular pole was blocked by pharmacological inhibition of the known MD-specific markers cyclooxygenase-2 (COX2) or neuronal nitric oxide synthase (NOS1), suggesting the key role of MD cells in this tissue remodeling process (Figure 1C). Fate tracking of MD cells for extended periods of time (>6 months) in either control or LS plus ACEi stimulation conditions using inducible MD-GFP mice resulted in no GFP-labeled cells outside of the MD area (data not shown), suggesting that MD cells themselves do not migrate out of their classic anatomical location.

### Intravital imaging of calcium signaling.

**Figure 2:**
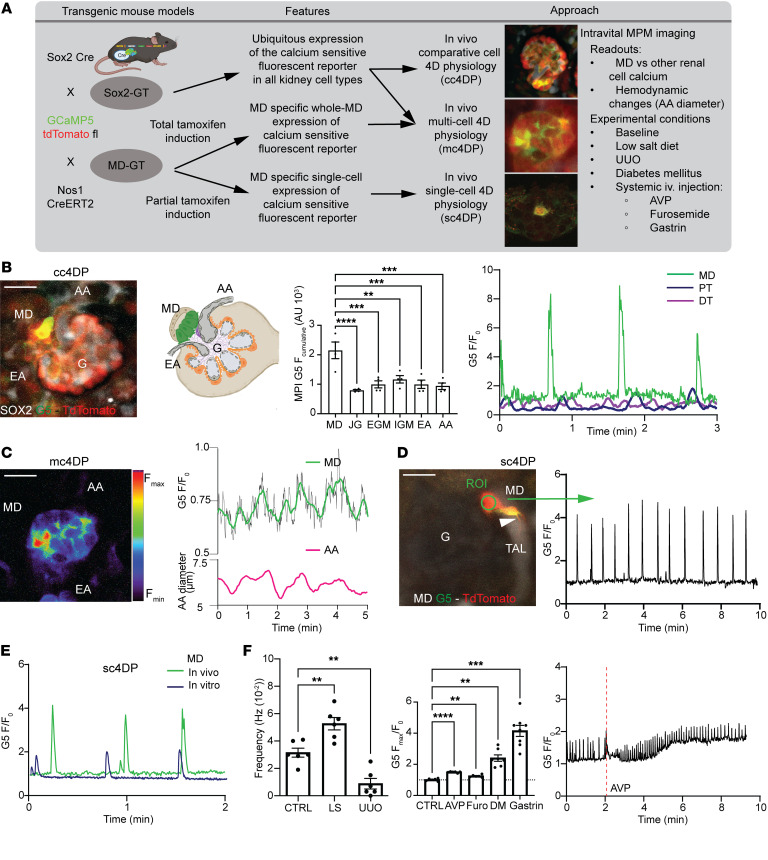
MD cell calcium signaling in vivo. (**A**) MPM imaging workflow to study 4D physiology in comparative (cc4DP), multicell (mc4DP), and single-cell (sc4DP) modes. Time-lapse recordings of same preparations are shown in Supplemental Video 2. (**B**) cc4DP mode using Sox2-GT mice. Left: Two-minute time-lapse maximum projection image (MPI) showing highest cumulative GCaMP5 (G5, green/yellow) fluorescence intensity (F) (reflecting cell Ca^2+^ elevations) in MD (green cells in attached glomerulus [G] drawing). Center: Comparison of G5F in single MD, juxtaglomerular (JG) renin, extra (EGM) and intraglomerular (IGM) mesangial cells, and afferent (AA) and efferent arteriole (EA) vascular smooth muscle cells, *n* = 4 (average of 5 cells/animal). Right: Time-lapse recordings of G5F normalized to baseline (F/F_0_) in MD (green), proximal tubule (PT, blue), and distal tubule (DT, purple) cells. (**C**) mc4DP mode using Sox2-GT mice. Left: Whole-MD pseudocolor MPI showing G5F heterogeneity between MD cells. Right: Simultaneous time-lapse recording of whole-MD G5F (green, smoothed) and AA diameter (magenta). (**D**) sc4DP mode using MD-GT mice. MD single cells show long axon-like processes (arrowhead, thick ascending limb [TAL] tubule fluid in gray scale) (left) and regular Ca^2+^ firing with 4-fold elevations in baseline (right). Scale bar: 20 μm. (**E**) MD single-cell G5F recordings in intact MD-GT mouse kidney in vivo and in vitro after isolation from freshly digested kidneys. (**F**) MD sc4DP responses to local/systemic stimuli. Left: MD cell Ca^2+^ firing frequency in control, low-salt diet (LS), and unilateral ureter obstruction (UUO). Center: Changes in MD G5F (baseline shown by dotted line) in response to systemically injected arginine-vasopressin (AVP), furosemide (Furo), gastrin, and in diabetes mellitus (DM). *n* = 6–8 (average of 4–5 cells/animal). Right: Time-lapse recording of whole-MD G5F during i.v. injection of the V1aR agonist arginine-vasopressin (AVP) given (indicated by red line) 2 minutes after recording baseline. Data represent mean ± SEM. **P < 0.01, ***P < 0.001, ****P < 0.0001, ANOVA followed by Dunnett’s test.

We next asked whether high signaling activity can be used as a clue to identify key cell type(s) that may drive local progenitors and kidney tissue remodeling similar to that in other organs. Therefore, intravital imaging of intracellular Ca^2+^ using MPM was performed in genetic mouse models to directly visualize the activity of all renal cell types or specifically MD cells in the intact living kidney cortex. We applied 3 main strategies to study cell physiology in 4 dimensions (4DP, in tissue volume over time) in comparative cell (cc4DP), multicell (mc4DP), or single-cell (sc4DP) modes as summarized in Figure 2A. To make comparisons to other renal cell types (in the cc4DP mode), Sox2–GCaMP5-tdTomato (Sox2-GT) mice were developed with ubiquitous expression of the genetically encoded calcium reporter GCaMP5, along with the calcium-insensitive tdTomato in all kidney cell types. Time-lapse intravital MPM imaging of Sox2-GT mice found robust, spontaneous Ca^2+^ transients in MD cells (Figure 2, B–F, and Supplemental Video 2). Among all cells at the glomerular vascular pole, including the highly contractile vascular smooth muscle cells of the glomerular arterioles, MD cells showed the highest cumulative elevations in Ca^2+^ by far (Figure 2B). MD cell Ca^2+^ signals did not conduct to adjacent contractile vascular cells, as evident from video recordings (Supplemental Video 2) and from the lower frequency of MD cell versus mesangial and vascular smooth muscle cell Ca^2+^ transients (Supplemental Figure 2A). In addition, the robust MD cell Ca^2+^ transients were autonomous (preserved in freshly isolated single MD cells, Figure 2E) and unique to MD cells in the renal cortex (based on comparison to other renal tubule epithelial cells (Figure 2B).

Intravital MPM imaging of the entire MD plaque (formed by ~25 individual MD cells) in horizontal optical sections (in the mc4DP mode) revealed considerable cellular heterogeneity, with individual MD cells featuring low, medium, or high Ca^2+^ activity (Figure 2C and Supplemental Video 2). Several, but not all MD cells in multiple plaque regions showed rapidly and laterally propagating Ca^2+^ responses, further suggesting the heterogeneity of cell-to-cell communication and coordination between individual MD cells (Supplemental Video 2). However, the robust Ca^2+^ signaling activity remained spatially confined to the MD plaque area and did not propagate to adjacent tubular or vascular segments (Supplemental Video 2). The whole-MD Ca^2+^ readout showed clustering of the single-cell Ca^2+^ transients and their regular oscillations over time (Figure 2C and Supplemental Figure 2C). Importantly, the whole-MD Ca^2+^ oscillations were simultaneous with the rhythmic changes in the diameter of the adjacent glomerular afferent arteriole, with the peak MD Ca^2+^ phase-matching the peak afferent arteriole diameter (vasodilatations) (Figure 2C).

To study the spatial and dynamic details of MD cell Ca^2+^ responses with truly single-cell resolution (in the sc4DP mode), MD-GT mice with MD cell–specific expression of GT were generated with a partial tamoxifen induction strategy, as used recently for single-cell expression and targeting (12). Consistent with the exclusively MD-targeting genetic strategy in the kidney using *Nos1-Cre* models (12, 20, 21), GT reporter expression was entirely specific for MD cells in the MD-GT mouse renal cortex (Supplemental Figure 2B) and illuminated the long, axon-like (Figure 2D and Supplemental Video 2), or multiple shorter basal cell processes of MD cells (Figure 2A). A few MD cells showed pacemaker-like regular Ca^2+^ oscillations, with the plateau showing approximately 4-fold elevations in baseline Ca^2+^ and an average frequency of 0.03 per second (Figure 2D). The average duration of single Ca^2+^ spikes (full width at half maximum) was approximately 2 seconds. MD cells responded to several diverse stimuli with altered steady-state Ca^2+^ and/or firing frequency, including altered tubular fluid composition (LS diet and the diuretic furosemide), mechanical strain (tubule flow), systemic neurohormone administration (arginine-vasopressin [AVP], gastrin), and metabolic states (diabetic hyperglycemia) (Figure 2F).

### MD cell transcriptome analysis.

**Figure 3:**
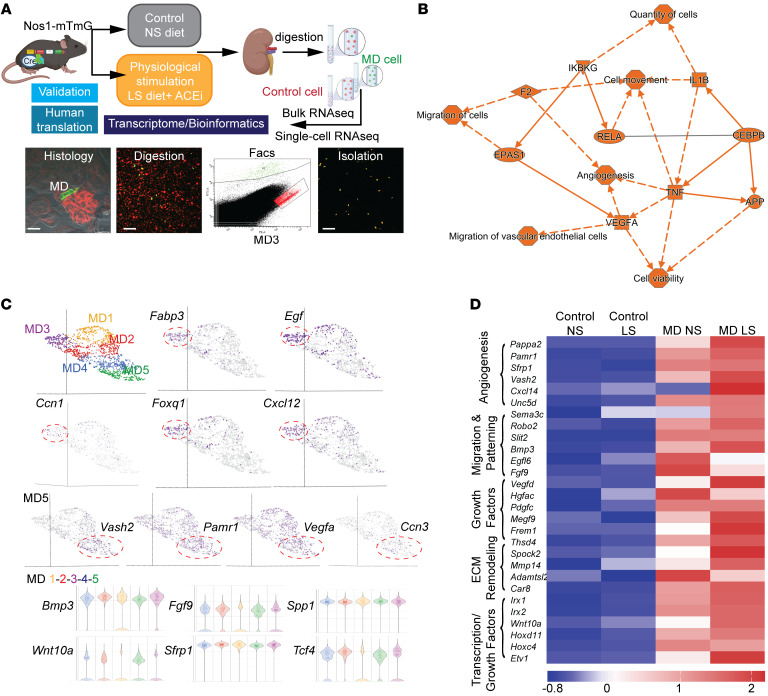
MD cell transcriptome analysis. (**A**) Workflow of MD and control cell isolation for transcriptome analysis in control (normal salt [NS]) and low-salt (LS) conditions. Scale bar: 25 μm. (**B**) Graphical summary of MD single-cell transcriptome analysis. The top activated (indicated by orange, positive *Z*-score) biological activities, pathways, and genes are listed based on unbiased IPA analysis. IPA system node shapes and colors are used (octagon, function; square, cytokine; triangle, kinase; ellipse, transcription regulator). (**C**) UMAP visualization (top) of integrated MD single-cell transcriptomic analysis from a single mouse in LS conditions. Graph-based analysis in Partek Flow identified 5 clusters (MD1–5). Top enriched genes (*Fabp3*, *Egf*, *Ccn1*, *Foxq1*, *Cxcl12*, *Vash2*, *Pamr1*, *Vegfa*, *Nov*) show clustering. Violin plot (bottom) of genes that are highly enriched in all 5 MD clusters. (**D**) Heatmap (mean expression) of top enriched MD-specific genes in MD vs. control cells in control (normal salt) and physiological stimulation (low salt + ACEi [LS]) conditions using bulk RNA analysis (*n* = 2 mice for MD, *n* = 4 mice for control cells from each condition). Genes were grouped into 5 categories as indicated according to their biological function. Scale indicates *Z*-score values.

**Figure 4:**
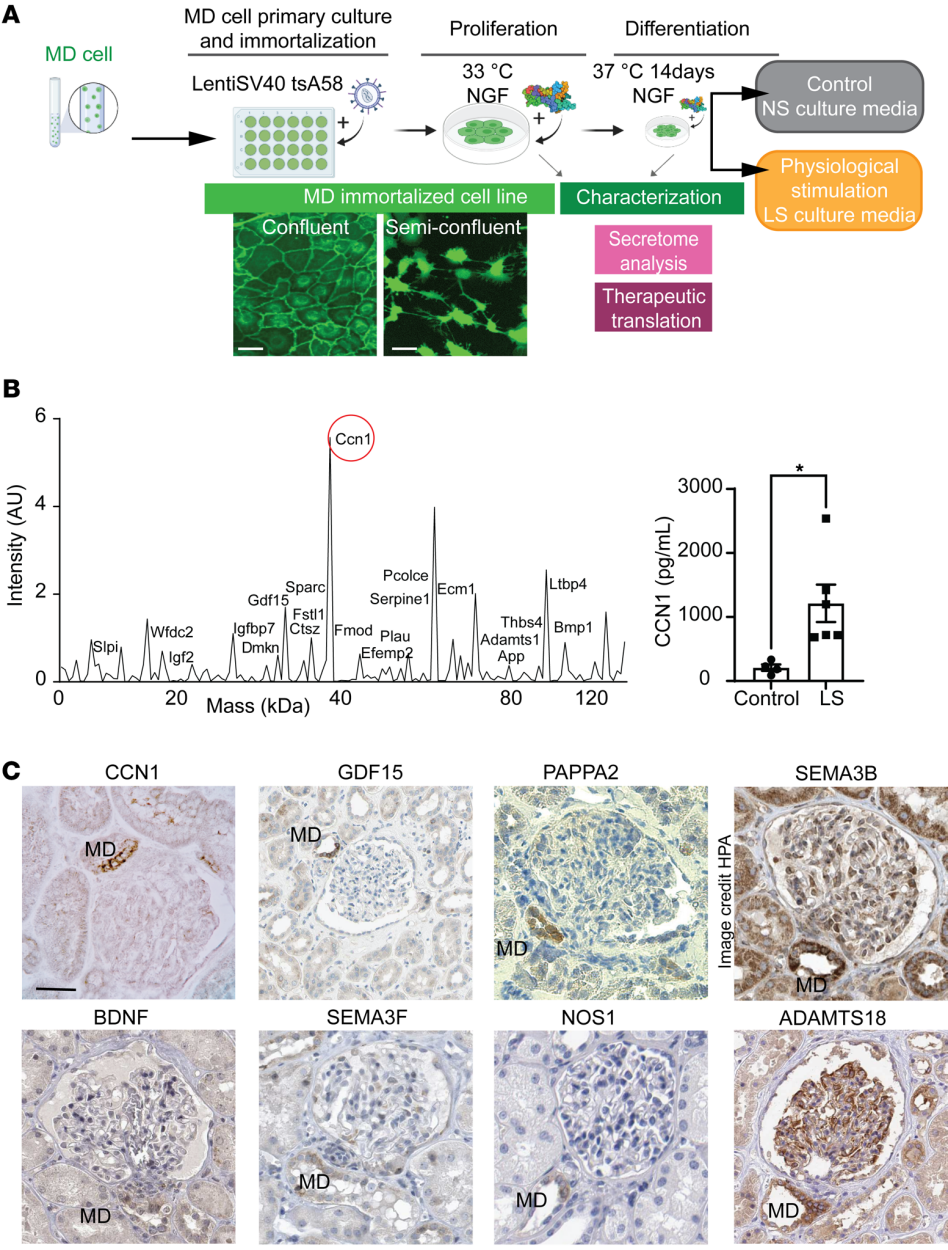
MD cell secretome analysis. (**A**) Workflow of the generation and characterization of the MD^geo^ cell line and its culturing (conditioning) in control (normal salt [NS]) and low salt (LS) conditions. Differentiated mMD^geo^ cells show an epithelial cobblestone-like pattern, while semiconfluent MD^geo^ cells feature long axon-like processes. Scale bar: 25 μm. (**B**) Mass spectrometry (left) and CCN1 ELISA (right) analysis of the LS-conditioned MD^geo^ cell culture medium. Mass spectrum plot representing the detected MD-derived secreted proteins in the MD^geo^ cell culture medium as indicated. LS, low-salt medium. Data represent mean ± SEM. *P < 0.05 with *t* test, *n* = 4–6. (**C**) Immunohistochemistry validation of the expression of top enriched mouse MD-specific genes or their homologous isoforms in the human kidney. Data are from the Human Protein Atlas (HPA) where indicated. Image available from https://www.proteinatlas.org/ENSG00000012171-SEMA3B/tissue/kidney Scale bar: 50 μm.

We next aimed to identify the molecular signature of MD cells in both control and physiological activation (LS) states with high-resolution using MD enrichment from MD-GFP mouse kidneys, as established and validated recently (12). The workflow included bulk and single-cell RNA-Seq and transcriptomic analysis (Figure 3A) and the generation of the immortalized MD cell line MD^geo^ (Figure 4A). A total of 28,000 MD cells (representing a minor fraction, ~0.2% of the total kidney cortical cell population) and 50,000 control cells from adjacent tubule segments were isolated from the cortex of freshly digested MD-GFP kidneys (*n* = 2 mice for MD and *n* = 4 mice for control cells from each condition) for bulk RNA isolation and transcriptome analysis. For single-cell RNA-Seq and transcriptome analysis, 894 and 1296 MD cells were analyzed from control and LS-induced conditions, respectively, each from a single MD-GFP mouse. Single-cell transcriptome analysis identified angiogenesis, cell movement, quantity and migration of cells, and migration of vascular endothelial cells as the major biological activities of MD cells (Figure 3B). Unsupervised graph-based clustering and UMAP visualization using Partek Flow revealed 5 subtypes of MD cells under both control and physiological activation (LS) conditions (MD1–5, Figure 3C). The high MD expression of specific growth and transcription factors and chemokines (*Fabp3*, *Egf*, *Ccn1*, *Foxq1*, *Cxcl12*) and angiogenic factors (*Vash2*, *Pamr1*, *Vegfa*, *Ccn3*) showed clustering, suggesting MD cell heterogeneity regarding their tissue remodeling regulatory functions. In contrast, all MD clusters were similarly enriched in other secreted cytokines, growth and extracellular matrix (ECM), and Wnt signaling factors (*Bmp3*, *Fgf9*, *Spp1*, *Wnt10a*, *Sfrp1*, *Tcf4*) that have well-known roles in controlling progenitor cells and tissue remodeling (Figure 3C). Bulk RNA-based MD transcriptomic analysis further confirmed the MD-specific expression of several angiogenic, cell migration and patterning, growth, ECM remodeling, and transcription factors. The expression of these factors was upregulated in physiological activation (LS) condition compared with controls (Figure 3D and Supplemental Table 1). The identified MD angiogenic factors included the CCN family of matricellular proteins, *Ccn1* (Figure 3C) and *Ccn3* (Figure 3C, ranked 38 highest enriched MD gene, Supplemental Table 1). The high-level expression of known MD cell markers (Supplemental Figure 3D and Supplemental Table 1, including *Pappa2*, *Nos1*, *Ptgs2* [COX2], *Slc12a1* [NKCC2]) validated the MD cell isolation and RNA-Seq approach. The high MD expression of neurohormone and metabolic receptors, such as the AVP (*Avpr1a*), gastrin and cholecystokinin (*Cckbr*), and glucagon receptor (*Gcgr*) (Supplemental Table 1), highlight the systemic sensory function of MD cells to maintain whole-body homeostasis and their role in interorgan crosstalk.

The analysis of control MD transcriptome (from mice on normal salt diet) confirmed the neuronal differentiation features of MD cells, in addition to their classic tubular epithelial characteristics (*Slc12a1*; *Slc9a2*, NHE2; *Kcnj1*, ROMK), Supplemental Figure 3D). Unbiased tissue specificity analysis using TissueEnrich (22) and the GTEx Multi Gene Query platform assigned a highly significant brain tissue identity to MD cells, while the transcriptome of control kidney cells showed high ranking of kidney specificity and no expression in brain (Supplemental Figure 3A). Canonical pathway analysis using IPA and Gene Set Enrichment analysis (Partek Flow) of MD cell transcriptome identified axon guidance as the most significant canonical pathway and relevant biological processes, such as synaptic transmission, synaptic plasticity, vesicle exocytosis, and membrane depolarization (Supplemental Figure 3, B and C). The MD transcriptome included high expression of Alzheimer’s disease risk genes (*App*, *Mapt*, *Bace1*, *Apoe*) and the nerve growth factor (NGF) receptor (*Ngfr*) (Supplemental Figure 3C). To further corroborate these findings, tissue volume rendering in 3D was performed from z-sections of optically cleared whole-mount MD-GFP kidneys, as established recently (20), and immunolabeled for endogenous MD-specific GFP expression and tyrosine hydroxylase (TH) or calcitonin gene–related peptide (CGRP), markers of sympathetic or sensory nerves, respectively. There was close anatomical association between MD cell basal processes and the sympathetic and sensory nerve endings, which together with the high expression of synaptophysin, a major synaptic vesicle protein specifically in MD cells and in nearby nerve endings (Supplemental Figure 3F) suggests that synaptic communications within the JGA involve the MD.

An immortalized mouse MD cell line named MD^geo^ was generated for in vitro cell biology studies by following a commonly used workflow, as illustrated in Figure 4A. Primary cultures of freshly isolated and sorted MD cells from MD-GFP kidneys were transfected with LentiSV40 tsA58 virus for temperature-sensitive proliferation (at 33°C) or differentiation (at 37°C) in the presence of NGF. As with the parent MD-GFP mouse model, the cultured MD^geo^ cell monolayer retained membrane-targeted GFP expression (Figure 4A). Confluent MD^geo^ cells had a cobblestone pattern typical of epithelial cells, while semiconfluent MD^geo^ cells featured long cell processes (Figure 4A). The MD phenotype of MD^geo^ cells was validated by the high expression of classic MD cell markers and their intact physiological function, including increased pERK1/2, COX2, and pp38 expression in response to LS culture conditions (Supplemental Figure 4, A and B) and NOS1-mediated NO release and COX2-mediated PGE_2_ production (Supplemental Figure 4C). Differentiated MD^geo^ cells had up to 10-fold higher expression of COX2 and NKCC2 compared with either undifferentiated MD^geo^ cells grown at 33°C or the formerly established but now extinct MD cell line MMDD1 (23) (Supplemental Figure 4B). Interestingly, the heterogeneity of the MD cell population was preserved in MD^geo^ cultures with cells showing variable size and shape, COX2 and NKCC2 expression, and NOS activity (Supplemental Figure 4). Mass spectrometry analysis of the LS conditioned MD^geo^ cell culture medium (MD secretome) identified the angiogenic matricellular protein CCN1 as one of the top enriched MD-secreted proteins (Figure 4B), confirming the transcriptome data (Figure 3C). MD conditioning by LS media resulted in a 6-fold increase in MD cell CCN1 secretion compared with control (Figure 4B).

The highly MD-specific expression of a selection of identified tissue remodeling genes (or their well-known, closely related homologs), *NOS1* (ranked the second highest enriched MD-specific gene, Supplemental Table 1), and the NGF homolog brain-derived neurotrophic factor (*BDNF*) was validated and translated to the human kidney on the protein level (Figure 4C and Supplemental Figure 3E).

### Manipulation of MD Wnt/β-catenin signaling.

**Figure 5:**
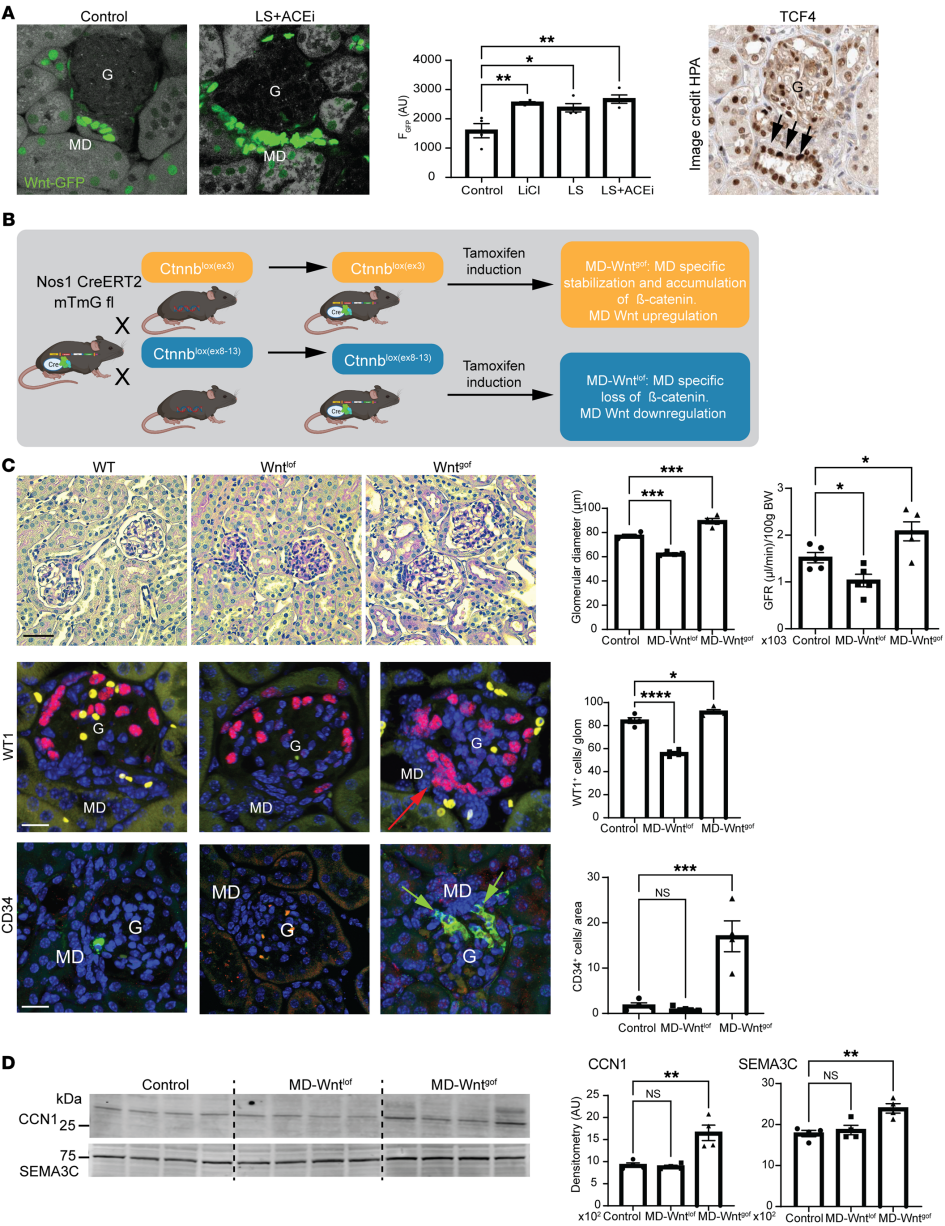
Manipulation of MD Wnt signaling alters glomerular structure and function. (**A**) Representative fluorescence images of frozen kidney sections (left) and quantification (center) of Wnt activity (GFP fluorescence [F] intensity, green) from mice with nuclear TCF/Lef:H2B-GFP reporter in control, LiCl (as positive control), LS, and LS+ACEi conditions; *n* = 4–6 (average of 5 MDs/animal). Intense GFP labeling in MD cells. Right: TCF4 immunohistochemistry in human kidney (data from the Human Protein Atlas [HPA]). Image available from https://www.proteinatlas.org/ENSG00000196628-TCF4/tissue/kidney Intense labeling in MD cells (arrows). G, glomerulus. (**B**) Illustration of the applied Cre/lox-based breeding strategies to generate inducible MD Wnt gain-of-function (MD-Wnt^gof^) and loss-of-function (MD-Wnt^lof^) mouse models. (**C**) Top: Renal histological (representative H&E images [left]) and functional (glomerular filtration rate [GFR, right]) features of MD-Wnt^gof^ and ^lof^ mice 2 months after tamoxifen induction; *n* = 4–5 (average of 5–10 glomeruli/animal). Note the enlarged or smaller cortical glomeruli in MD-Wnt^gof^ and ^lof^ mice, respectively, compared with control WT mice, with extracellular (mesangial) matrix accumulation in MD-Wnt^lof^ mice. Bottom: Representative immunofluorescence images (left) and statistical summary (right) of WT1^+^ (red) and CD34^+^ (green) cell number. Note the high cell density at the macula densa (MD) cell base (arrows). Yellow (in WT1) and orange (in CD34) colors represent the autofluorescence of red blood cells. Scale bars: 25 μm. Nuclei are labeled blue with DAPI. (**D**) Altered expression of MD-specific proteins in renal cortical homogenates, including CCN1 and SEMA3C; *n* = 4. Data represent mean ± SEM. *P < 0.05, **P < 0.01, ***P < 0.001, ****P < 0.0001, ANOVA followed by Dunnett’s test.

To confirm the tissue remodeling function of MD cells in vivo, we next used MD-specific genetic manipulation of Wnt signaling and studied the resulting renal phenotype. Wnt activity is high in MD cells, and it further increases with LS stimulation (Figure 5A). Inducible MD Wnt loss-of-function (MD-Wnt^lof^) and gain-of-function (MD-Wnt^gof^) mouse models were generated (Figure 5B) and validated by the MD-specific decreased or increased expression of the genetic WntGFP reporter and the Wnt target *Axin2*, respectively (Supplemental Figure 5A). Blood pressure, kidney and body weight, and albuminuria were normal (Supplemental Figure 5B). MD-Wnt^gof^ induction in adult mice resulted in enlarged glomeruli and increased glomerular filtration rate (GFR) within 6–8 weeks (Figure 5C). In contrast, MD-Wnt^lof^ induction resulted in smaller glomeruli and reduced GFR (Figure 5C). The number of WT1^+^ mesenchymal progenitor cells/podocytes and CD34^+^ endothelial precursor cells (alternatively Meis2^+^ [endothelial marker], ref. 24, and Ren^+^ [mesenchymal marker] cells, respectively; Supplemental Figure 5C) at the glomerular vascular pole increased in a MD-centric pattern in MD-Wnt^gof^ mice, while these cell numbers were reduced or unchanged in MD-Wnt^lof^ mice (Figure 5C and Supplemental Figure 5C). MD-Wnt^gof^ increased the expression of CCN1 and other secreted angiogenesis promoting factors, including SEMA3C, that are known Wnt target genes (25) (Figure 5D). Altogether, these results suggest that MD cells via Wnt signaling are physiologically and functionally important regulators of renal progenitor cells and tissue remodeling.

### Key role of NGF/NGFR signaling in MD cell and kidney functions.

Since *Ngfr* was the highest expressed growth factor receptor in MD cells (ranked 64th most-enriched MD gene; Supplemental Table 1), we tested the functional importance of NGF and NGFR in MD cells in vitro and in the kidney in vivo. Immunofluorescence analysis validated the transcriptome data and confirmed the MD cell–specific expression of the p75NTR NGFR in the mouse kidney and in MD^geo^ cells, including in the cell membrane (Supplemental Figure 6A). NGF significantly increased cell proliferation (Supplemental Figure 6B) and was absolutely essential for the long-term survival of MD^geo^ cells in culture (data not shown). NGFR expression was significantly increased by the LS culturing condition in MD^geo^ cells (Supplemental Figure 6C), suggesting the physiological relevance of NGFR in MD cells. NGF treatment significantly increased the expression of the classic and functionally key MD cell markers COX2, NKCC2, and NOS1 and induced phosphorylation of ERK1/2 MAP kinases, IKB, and TrkA-B, but not Akt in a variable time-dependent manner (Supplemental Figure 6, C and D). Using NGF-GFP reporter mice and amyloid precursor protein (APP) immunohistochemistry, the expression of potential NGFR ligands NGF and soluble APP was confirmed in nearby renin producing juxtaglomerular (JG) and collecting duct intercalated cells (Supplemental Figure 6A).

**Figure 6:**
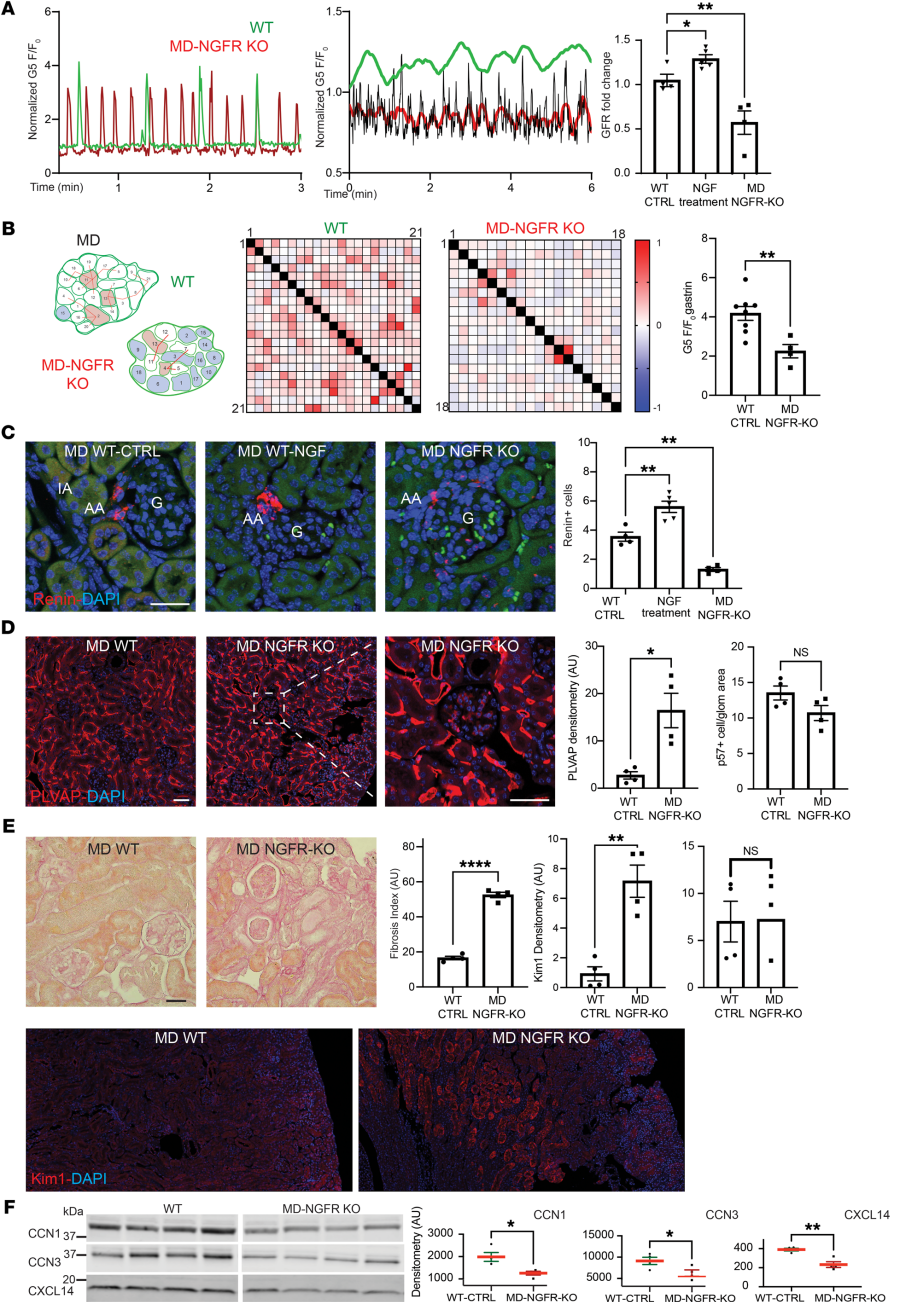
The phenotype of MD-NGFR–KO mice. (**A**) Increased frequency of MD cell Ca^2+^ transients in MD-NGFR–KO vs. WT mice. Single MD cell (left) and whole-MD recording (center, original/smoothed) of G5F transients in WT (green) and MD-NGFR–KO mice (red). GFR changes (normalized to baseline before induction/treatment) in WT treated with vehicle (control) or NGF and MD-NGFR–KO mice (right); *n* = 4–5. (**B**) Reduced MD cell connectivity and sensitivity in MD-NGFR–KO vs. WT mice. Left: Functional MD cell–to-cell connectivity map of all 21 (WT) and 18 (MD-NGFR–KO) individually numbered MD cells. Red line connecting individual cell pairs indicates Pearson’s *r* > 0.35. Red and blue cell color indicates hub and lone cells, respectively. Center: Heatmap of each MD cell pair’s Pearson’s coefficient in 2-color gradient, as in scale. Right: Effect of i.v. gastrin on MD cell Ca^2+^ (G5F) in WT vs. MD-NGFR–KO mice; *n* = 4–8 (average of 4–5 MD cells/animal). (**C**–**E**) Renin cell density (**C**), endothelial injury and podocyte number (**D**), and renal pathology (**E**) in WT mice treated with vehicle (control) or NGF,and MD-NGFR–KO mice. *n* = 4–5 (average of 5 glomeruli/animal). Renin, PLVAP, KIM1 immunofluorescence (red) images and PAS-stained kidney tissue sections, and summary of respective cell numbers, labeling density (tissue fibrosis index), and albuminuria (ACR). Cell nuclei are labeled blue with DAPI, tissue autofluorescence (**C**, green) is shown for tissue morphology. Scale bar: 50 μm (**C**–**E**). (**F**) Representative immunoblots and summary of MD-specific protein expression in renal cortical homogenates, including CCN1, CCN3, and CXCL14 in WT vs. MD-NGFR–KO mice; *n* = 4. Data represent mean ± SEM. *P < 0.05, **P < 0.01, ***P < 0.001, ****P < 0.0001 using Student’s *t* test (**B** and **D**–**F**) or ANOVA followed by Dunnett’s test (**A** and **C**).

We next developed mice with inducible, MD-specific knockout of NGFR (MD-NGFR–KO) to examine the functional role of MD cell NGFR signaling in vivo, 4 weeks after tamoxifen induction. Immunofluorescence localization and renal cortical tissue immunoblotting validated the successful MD-specific deletion of the p75NTR NGFR in MD-NGFR–KO mice (Supplemental Figure 6, A and C). Compared with that of WT mice, the frequency of MD cell Ca^2+^ transients increased 4-fold in MD-NGFR–KO mice (Figure 6A). In contrast to regular periodic oscillations in WT mice (Figure 2C), MD-NGFR–KO mice featured irregular chaotic-like oscillations in whole-MD Ca^2+^ (Figure 6A). A cell-to-cell functional connectivity map (MD connectome) with preserved MD architecture and a heatmap were generated based on Pearson’s correlation analysis of time-lapse Ca^2+^ recordings between all MD cell pairs in WT and MD-NGFR–KO mice (Figure 6B). Cell-to-cell connectivity and sensitivity of MD cells were greatly reduced in MD-NGFR–KO mice compared with that in WT mice (Figure 6B). The connectivity map of WT MD plaque identified 3 “hub” cells within the whole MD that had the highest number of connections, and 1 “lone” cell that had 0 connections (Figure 6B). In the MD of MD-NGFR–KO kidney, there were only 2 small “hub” cells within the whole MD, while a great number of “lone” cells were identified that had no connections to other MD cells (Figure 6B). NGF treatment for 1 week significantly increased the number of renin-producing juxtaglomerular (JG) cells and GFR in WT mice, while renin cell number and GFR were significantly lower in MD-NGFR–KO mice (Figure 6, A and C). MD-NGFR–KO mice featured glomerular endothelial injury (based on increased plasmalemmal vesicle associated protein [PLVAP] expression) (26), kidney injury (based on KIM1 expression), and tissue fibrosis (Figure 6, D and E) and showed accumulation of the classic neurodegeneration marker p-tau (S199) in MD cells (Supplemental Figure 6A). Albuminuria and podocyte number were normal at this time point (Figure 6, D and E). The expression of MD-specific angiogenic and tissue remodeling factors CCN1, CCN3, and CXCL14 was significantly reduced in MD-NGFR–KO mice compared with that in WT mice (Figure 6F).

### Human translation.

**Figure 7:**
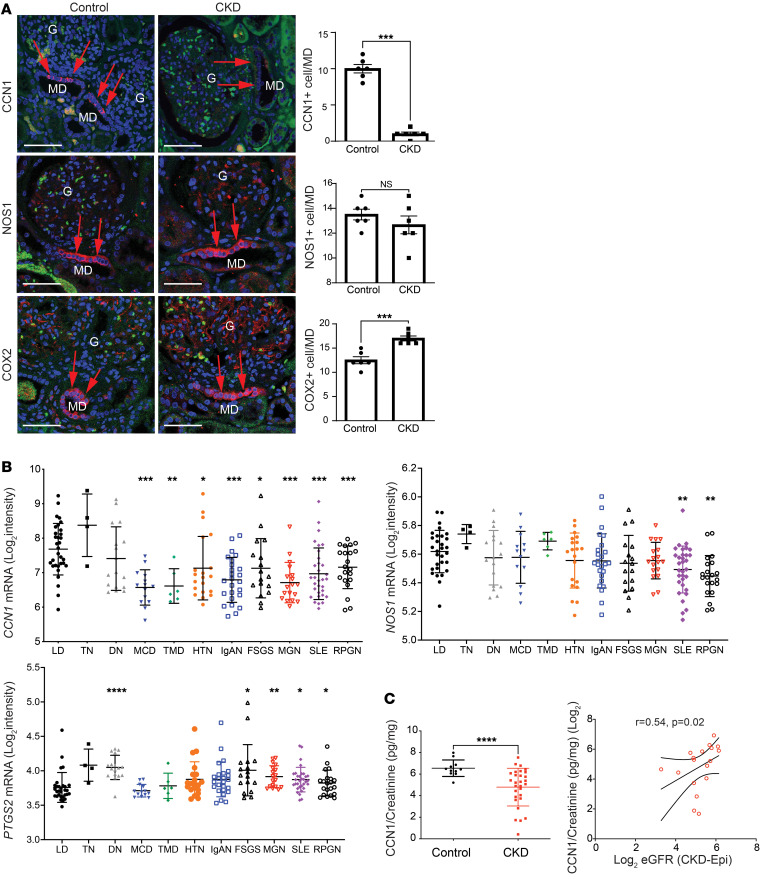
CCN1 expression in the kidney in patients with normal kidney function or CKD. (**A**) Immunofluorescence labeling (red, left) and quantification (right) of MD cell markers CCN1 (top), NOS1 (center), and COX2 (bottom) in human kidney sections. Note the strong CCN1 expression exclusively in cells of the macula densa (MD; red arrows) in controls and mostly absent labeling in kidney tissue samples from patients with CKD, in contrast to the pattern in NOS1 and COX2 labeling; *n* = 6 (average of 5 MDs/sample). Nuclei are labeled blue with DAPI; green is tissue autofluorescence. G: glomerulus. Scale bars: 25 μm. (**B**) Intrarenal *CCN1* (CYR61), *NOS1*, and *PTGS2* (COX2) mRNA expression in kidney biopsies (tubulointerstitial compartment) from living donor (LD), tumor nephrectomy (TN), and patients with CKD with various etiologies from the ERCB. LD (*n* = 31), TN (*n* = 4), diabetic nephropathy (DN, *n* = 17), minimal change disease (MCD, *n* = 14), thin basement membrane disease (TMD, *n* = 6), arterial hypertension (HTN, *n* = 20), IgA nephropathy (IgAN, *n* = 25), focal segmental glomerulosclerosis (FSGS, *n* = 17), lupus nephritis (SLE, *n* = 32), membranous glomerulonephropathy (MGN, *n* = 18), and vasculitis (RPGN, *n* = 21). Differential expression comparison between LD and each disease subtype was performed using *t* test. (**C**) The association of urinary CCN1 levels with kidney function. Comparison of urinary CCN1 in individuals acting as controls (*n* = 11) and patients with CKD (*n* = 29) (left) and the positive correlation between urinary CCN1 excretion and eGFR in patients with CKD; *n* = 18, log_2_-transformed urinary CCN1/creatinine ratios are shown (right). Data represent mean ± SEM. *P < 0.05, **P < 0.01, ***P < 0.001, ****P < 0.0001 using Student’s *t* test.

To study the relevance of the MD tissue remodeling function to the human condition and CKD, we first analyzed the expression of the angiogenic factor CCN1 in freshly nephrectomized and fixed human kidney tissues from patients with normal kidney function or CKD (estimated GFR [eGFR], <50 mL/min/1.73 m^2^). CCN1 protein expression was entirely MD specific in normal human renal tissues (Figure 7A). The number of CCN1^+^ MD cells was significantly reduced to almost undetectable levels in CKD, while the total MD cell number was maintained or even increased based on NOS1 and COX2 immunolabeling (Figure 7A).

Transcriptomic data in human kidney biopsies from the European Renal cDNA Biobank (ERCB) were analyzed to further investigate the relevance of *CCN1* to human CKD. Compared with living donors, *CCN1* was significantly underexpressed in patients with CKD with minimal change disease, thin basement membrane, membranous glomerulonephropathy, IgA nephropathy, lupus nephritis, vasculitis, hypertensive nephropathy, and focal segmental glomerulosclerosis (FSGS) (Figure 7B). *CCN1* was within the top 1% of underexpressed genes in CKD. Reduced expression in CKD was specific for *CCN1*, in contrast to the classic MD cell marker genes *NOS1* and *PTGS2* (Figure 7B).

In addition, we tested whether CCN1 can be detected in human urine samples and if urinary CCN1 levels correlate with kidney function, thus making CCN1 a potential biomarker. The results confirmed the presence of detectable levels of urinary CCN1 excretion, and urinary CCN1 was significantly diminished in patients with CKD (4.8 ± 0.3 pg/mg CCN1/creatinine ratio) compared with that of volunteer controls (6.5 ± 0.2 pg/mg CCN1/creatinine ratio) (Figure 7C). Urinary CCN1/creatinine levels showed a significant positive correlation with eGFR (Figure 7C), suggesting that reduced urinary levels of CCN1 are associated with loss of kidney function. In addition, patients with lower CCN1/creatinine levels (defined as less than average value among this group of patients) showed a trend of having lower eGFR and older age, and they were more likely to progress to end-stage kidney disease (data not shown).

### Therapeutic use of MD-secreted factors.

**Figure 8:**
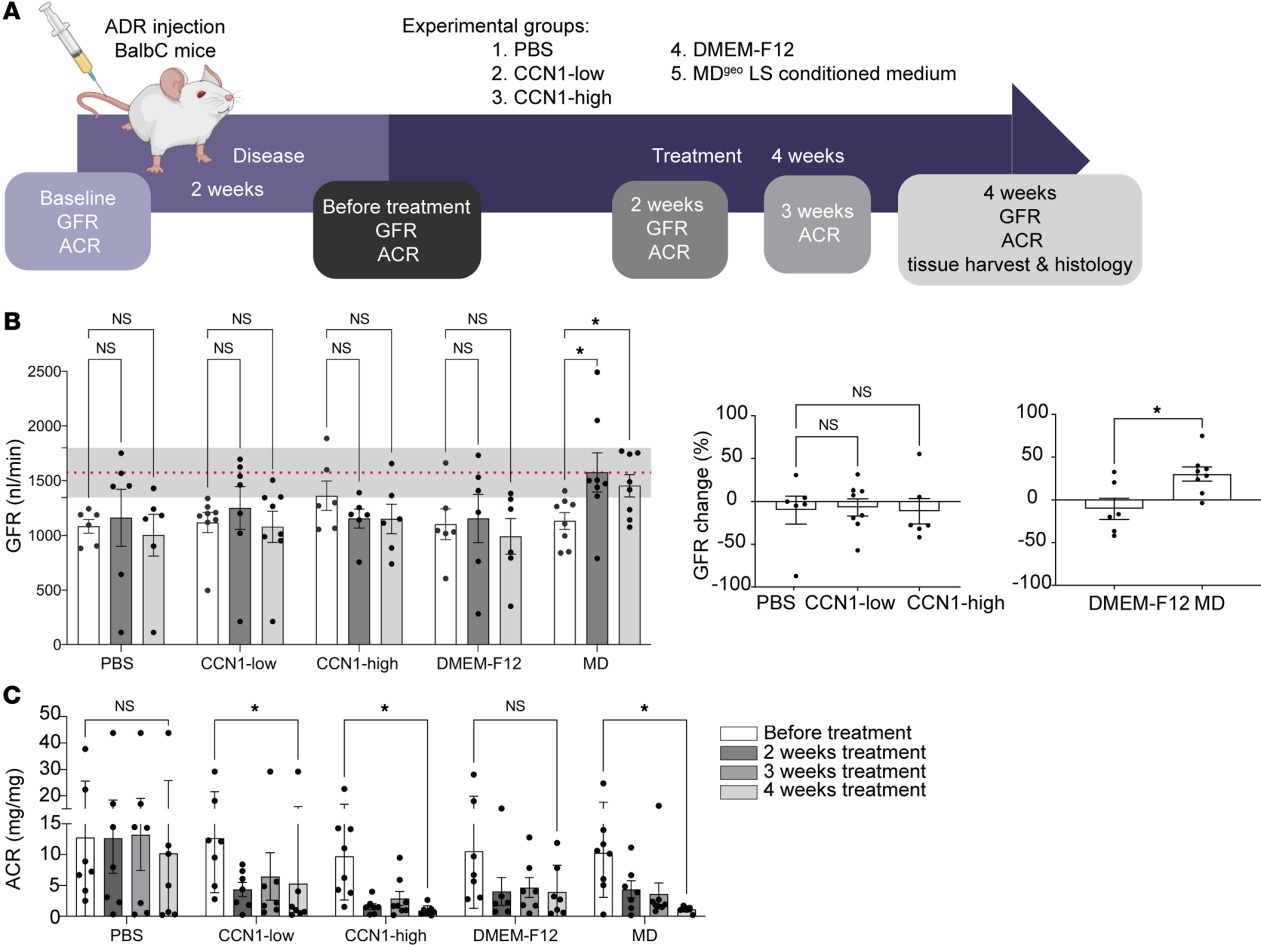
Treatment with MD biologicals improves kidney function in CKD. (**A**) Illustration of therapeutic study design for testing the effects of MD biologicals (human recombinant CCN1 and LS-conditioned MD^geo^ cell culture media) using the adriamycin (ADR) model of glomerulosclerosis in BALB/c mice. (**B**) Time course of the absolute (left) and relative (normalized to baseline before treatment, right) changes in GFR followed in the same mice measured by the MediBeacon noninvasive transcutaneous method. Note the significant improvement of GFR returning to normal baseline levels (the red dotted line represents mean ± SEM [gray shaded area], measured at baseline) in the MD treatment group indicating functional regression of FSGS pathology; *n* = 6–8. (**C**) Time course of albuminuria (albumin/creatinine ratio [ACR]) changes followed in the same mice measured by ELISA. Note the significant improvement in albuminuria in the CCN1 and MD treatment groups in contrast to the PBS and DMEM-F12 controls; *n* = 6–8. Data represent mean ± SEM. *P < 0.05, ****P < 0.0001, 2-way (mixed-effect) ANOVA with Tukey’s test (**B**, left, and **C**), 1-way ANOVA followed by Dunnett’s test (**B**, center), or *t* test (**B**, right).

**Figure 9:**
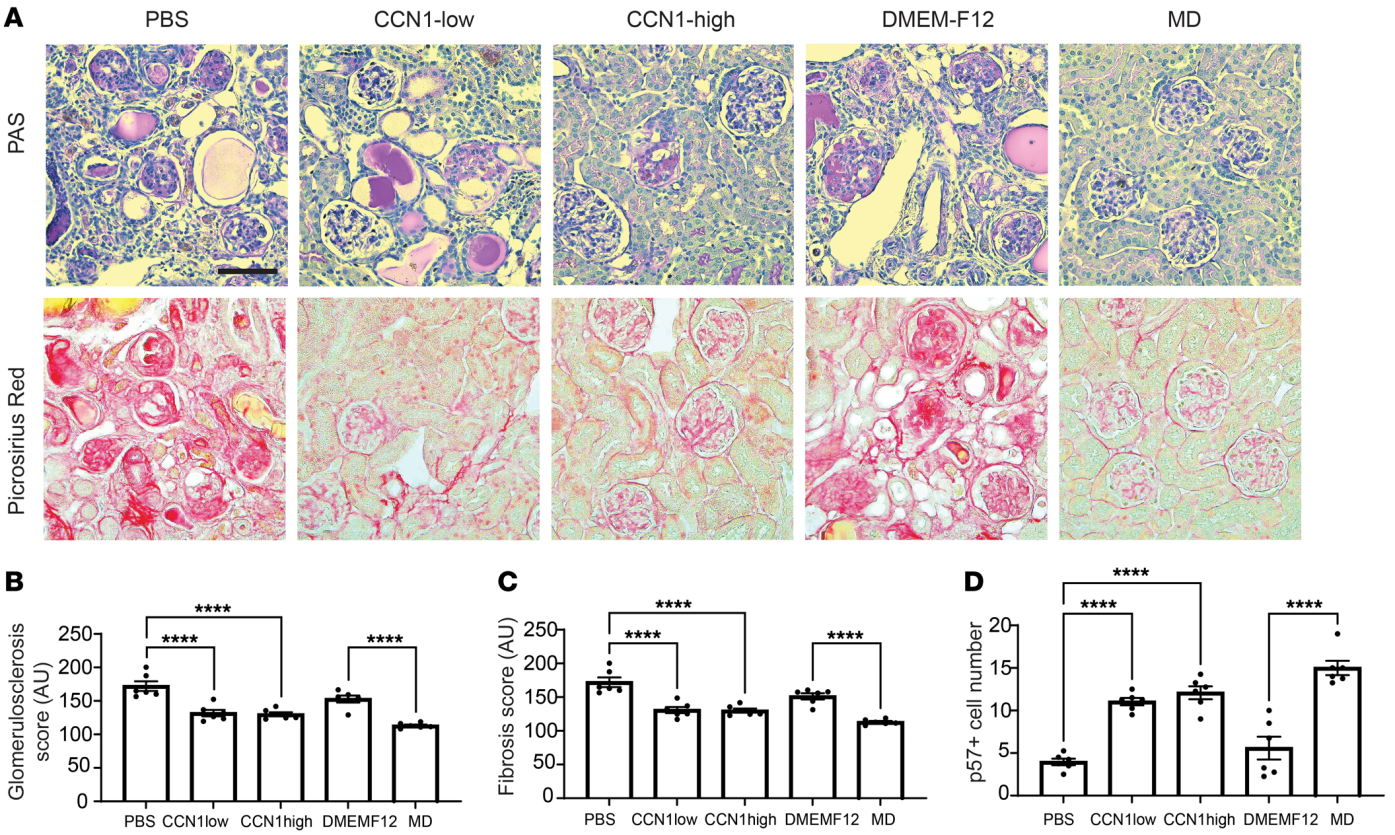
Treatment with MD biologicals improves kidney structure in CKD. (**A**) PAS- (top row) and Picrosirius Red–stained (bottom row) histology images of kidney tissues harvested at the end of treatment in all treatment groups. Scale bar: 50 μm. (**B**–**D**) Quantification of glomerulosclerosis (**B**), tubulointerstitial fibrosis (**C**), and p57^+^ cell number (podocyte preservation) (**D**) in all treatment groups; *n* = 6 (average of 5 areas/animal). Data represent mean ± SEM. ****P < 0.0001, ANOVA with Šidák’s test.

The effects of MD-derived biologicals were tested in the robust CKD model of adriamycin-induced (ADR-induced) FSGS in BALB/c mice (Figure 8A). A single ADR injection induced severe glomerulosclerosis (GS) pathology after 2 weeks, indicated by reduced GFR and high-level albuminuria (Figure 8, B and C). At this point, treatment of the preexisting GS was initiated using one of the following 5 biologicals: saline (PBS), recombinant CCN1 in low or high dose, control DMEMF12 culture medium, or LS-conditioned cell culture medium of the MD^geo^ cell line (Figure 4, A and B). Animals were followed-up for 4 weeks of treatment. Mass spectrometry analysis and CCN1 ELISA of the conditioned MD^geo^ cell culture medium confirmed the secretion of MD cell factors and informed the therapeutic dose of low CCN1 (Figure 4B), while high-dose CCN1 was chosen based on previous work in liver (27). In contrast to control PBS or DMEMF12 medium which had no effect, treatment with CCN1 (either with low or high dose) or LS-conditioned MD medium caused strong improvements in albuminuria (Figure 8C). In contrast to all other groups, treatment with LS-conditioned MD medium improved GFR, which returned to normal baseline levels, indicating functional regression of FSGS pathology (Figure 8B). Subsequent kidney histological analysis showed severe GS and tissue fibrosis in control PBS- and DMEMF12-treated groups, while CCN1 or LS-conditioned MD medium treatments greatly improved FSGS histopathology, p57^+^ podocyte number, and tubulointerstitial fibrosis (Figure 9).

## Discussion

This study uncovered what we believe are new and molecular mechanisms of endogenous kidney tissue remodeling controlled by the understudied minority cell type of the MD. The activation of these MD tissue remodeling mechanisms by organ-specific physiological stimuli (e.g., LS diet) rather than pathological tissue injury (Figure 1B and Supplemental Figure 1A) is an important finding that has strong foundations in evolutionary biology, physiology, clinical nephrology, and recent developments in regenerative biology. The loss and conservation of body fluid and salt is a known major driver of the evolution of the mammalian nephron (8), major physiological adaptations to increase kidney function and maintain body fluid homeostasis (10), and in the protective effect of low dietary salt intake (and ACEi) to slow down CKD progression (28, 29). The identified cell structural (signal propagation via axon-like processes; Figure 2D) (12), functional (calcium signaling, local and systemic sensing; Figure 2, B–F), and molecular features (tissue remodeling factors, CCN1; Figure 3), including neuronal differentiation (NGFR; Supplemental Figure 3) suggest that the MD is a much more complex and key sensory and regulatory cell type in the nephron than previously thought (9). The neuronal differentiation of the MD tubular epithelium and the role of MD cells in tissue remodeling are in line with the recently identified role of peripheral neuronal mechanisms in local tissue regeneration of other organs (14–16). Pharmacological (COX2 and NOS1 inhibitors; Figure 1C) and genetic approaches (MD-Wnt^gof/lof^ mice, Figure 5, and MD-NGFR–KO mice, Figure 6) confirmed MD specificity and validated the physiological and functional importance of the MD tissue remodeling mechanisms. Overall, the present study provided important mechanistic insights into MD function, which informed preliminary clinical and therapeutic translation and confirmation of the therapeutic benefit of MD biologicals for CKD.

MD cells are main salt sensors and regulators of kidney function, but our traditional knowledge of these unique cells has been limited to their role in maintaining renal hemodynamics and activity of the renin-angiotensin system (10). MD cell MAPK (20), mTOR (21), and the presently identified calcium, Wnt, and Ngfr signaling activities are distinctively high compared with other renal cell types. These MD cell signaling pathways are further stimulated by physiological activation of the MD (LS diet) and play key roles in the neuronal differentiation and tissue remodeling functions of MD cells (Figures 2–6 and Supplemental Figure 6). These results shed light on the importance and complexity of MD sensory and regulatory mechanisms. In contrast, high-salt diet, which is a known renal and cardiovascular disease risk factor (30), had a negative effect on NG2^+^ cell recruitment (Supplemental Figure 1A), suggesting that elements in the unhealthy diet (e.g., high dietary salt intake) may blunt MD tissue remodeling mechanisms. The applied LS and ACEi treatment is known to be associated with moderate reduction in blood pressure that is well within the autoregulatory range (20) and did not cause kidney injury in the present study (Supplemental Figure 1A). The results of our comprehensive in vivo pharmacologic, genetic, therapeutic, and in vitro cell approach strongly suggest the importance of MD-specific mechanisms in LS and ACEi–induced tissue remodeling, rather than the indirect effects of reduced blood pressure.

Serial intravital MPM imaging (17–19, 31) provided critically valuable visual clues regarding the dynamic (within 2 weeks) and the MD-centric pattern of the recruitment and clonal propagation of vascular pole progenitor cells (Figure 1B and Supplemental Figure 1, D and E). The accumulation of renin^+^, WT1^+^ and CD34^+^ cells (known mesenchymal/podocyte and endothelial progenitor cell markers, respectively, refs. 31–34) at the glomerular vascular pole under MD stimulation conditions (Supplemental Figure 1, D and E, and Figure 5C) strongly suggested the MD secretion and paracrine actions of local tissue remodeling factors that control the activation, recruitment, and differentiation of resident progenitor cells. These findings expand on a wealth of previously published work that characterized the effects of LS and ACEi to cause glomerular and vascular remodeling via a combination of MD and JG cell mechanisms, including the induction of progenitor cell populations and the inhibitory effects of COX2 inhibitors (35–37). Vascular pole endothelial progenitors and cells of the renin lineage have been identified as local progenitor cells for glomerular cell types (19, 34), including the role of WT1 in the proliferation, migration, and differentiation of the renin lineage toward podocyte fate (31). The MD point-source and 3D-structured tissue remodeling mechanism may be involved in the previously described progenitor/podocyte cell marker gradient (38) and podocyte precursor cell accumulation in the vascular pole region of Bowman’s capsule, including at young age (39, 40) and in response to ACEi treatment (41). COX2 and NOS1 dependence of mesenchymal and endothelial progenitor cell recruitment (Figure 1C) is consistent with the role of MD cell factors, including PGE_2_, which was shown to activate the recruitment and differentiation of CD44^+^ mesenchymal progenitors to the renin cell fate (37). MD mechanisms may be involved in the protective effects of selective COX2 inhibition in diabetic kidney injury (at least in the early hyperfiltration phase), which is associated with MD gain of function, mesangial expansion, and vascular pole angiogenesis (42–44).

Studying in vivo sc4DP of MD cells using MPM imaging (Figure 2) uncovered interesting functional features, including autonomous, rhythmic Ca^2+^ oscillations, suggesting pacemaker function, and the coordination and clustering of single MD cell Ca^2+^ transients into a cumulative whole-MD signal with a frequency (0.03 Hz) that matches TGF oscillations (45). NOS1 and COX2 enzymes that generate the vasodilators NO and PGE_2_ are known classic MD-specific markers in the renal cortex (10, 11, 46) and known to be Ca^2+^ sensitive (47, 48). MD Ca^2+^ activity increased in response to stimuli that cause LS in the tubular fluid microenvironment, including LS diet and the diuretic furosemide that inhibit MD NaCl entry via NKCC2 (*Slc12a1*) (Figure 2F). This is consistent with the classic function of MD cells in tubular fluid salt sensing that is known to involve NKCC2 and MD cell depolarization in response to altered luminal [NaCl] (9, 49). The presently identified MD cell functions clearly require future follow-up studies that will help to better understand the complexity of renal blood flow regulation. Importantly, these sc4DP imaging studies illuminated features of MD cells that are consistent with their role in tissue remodeling. The present results confirm the recently reported expression of neuronal genes in MD cells (13) and further characterize the MD as a neuronally differentiated modified epithelium, with special sensory and regulatory functions.

Until the present MD enrichment approach, only a few studies reported MD transcriptome data using a limited number of cells (13, 50–52). The top MD-enriched and LS-activated angiogenic (*Pappa2*, *Pamr1*, *Sfrp1*, *Vash2*, *Vegfa*, *Ccn1*), cell migration and patterning (*Unc5d*, *Sema3c*, *Robo2*, *Slit2*), growth (*Bmp3*, *Egf*, *Fgf9*), and ECM signaling and remodeling (*Frem1*, *Thsd4*, *Mmp14*, *Adamtsl2*) factors and gene networks, cytokines, and chemokines (Figure 3) have well-defined roles in angiogenesis, progenitor cell recruitment and differentiation, ECM and tissue remodeling, and regeneration of several organs (53–55). These results provide molecular-level insights and confirmation of the intravital progenitor cell–tracking data (Figure 1) and MD tissue remodeling functions. The MD secretion of several of these factors including the antifibrotic and proangiogenic CCN1 was confirmed in the present study (Figure 4B). The altered synthesis of at least some of these MD factors in MD-Wnt^gof^ mice and MD-NGFR–KO mice (Figure 5D and Figure 6F) and the resulting tissue structural alterations further suggest their importance in MD-regulated tissue remodeling.

Wnt signaling and CCN1 have well-known roles in tissue and organ remodeling and repair (54, 56), but also in inflammation, acute kidney injury, and other pathology development (57, 58), suggesting their context-dependent functions. NGF signaling via the p75NTR is another key MD cell signaling pathway identified in the present study that appears to be playing dual roles in MD cell neuronal differentiation and MD-directed tissue remodeling. In addition to high NGFR expression, the TrkA/B NGF receptors are also expressed in MD cells (Supplemental Figure 6, A–D). Future investigation is needed regarding the complexity of MD cell NGF signaling, including the identification of receptor subtypes and agonists (59). JG renin cells, collecting duct intercalated cells, and a subset of MD cells produce neurotrophins, including NGF, sAPPα (60) (Supplemental Figure 6A), and the related BDNF (Figure 4C). This suggests bidirectional multicellular crosstalk between MD and other JG cells that are known to form a classic physiological functional unit with MD cells (10, 20, 61). Interestingly, NGFR deficiency triggered MD hyperactivity at the single-cell level (increased Ca^2+^ firing frequency, Figure 6A), which may have been due to hyperexcitability or inhibitory dysfunction, which are well-known pathogenic events in neurodegeneration (62). The loss of cell-to-cell connectivity and lack of oscillating vasomotor (TGF) signal (Figure 6, A and B) were associated with reduced hemodynamics (GFR decline), decreased renin cell number, and renal tissue injury and fibrosis (Figure 6, A–E), which are classic features of CKD. The irregular chaotic oscillations in whole-MD Ca^2+^ in MD-NGFR–KO mice (Figure 6A) were reminiscent of the previously reported chaotic oscillations in MD-mediated TGF in different models of hypertension (63, 64), suggesting the pathogenic role of the MD cell network when dysregulated. The structural and functional heterogeneity of individual MD cells (Figure 2, B and C) (12, 65) was reflected in their gene profile (Figure 3C). The detailed characterization of the MD cell subclusters (Figure 3C) and whether their calcium signaling activity (pacemaker, or highly connected hub cells, or lone cells, as shown in Figure 2C and Figure 6B) corresponds to a specific tissue remodeling activity and/or 3D spatial localization (afferent/efferent arteriole or mesangium-facing localization) requires further study.

Initial clinical and therapeutic translation of the presently identified MD mechanisms found that CCN1 was specifically expressed in MD cells in the human kidney, and its expression was reduced in nondiabetic CKD based on both mRNA and protein levels (Figure 7, A and B). These changes were specific for CCN1 and not observed for other MD cell markers, like NOS1 and PTGS2 (COX2, Figure 7, A and B), indicating transcriptional regulatory mechanisms rather than reduced MD cell number as the underlying cause. In addition, CCN1 was detectable in human urine samples and correlated with glomerular function (Figure 7C), suggesting potential utility and need for further development of MD-derived urinary CCN1 as a noninvasive biomarker of glomerular structure and function and CKD progression. It should be noted that patients with CKD are usually counseled to maintain a LS diet and take ACEi. Therefore, the reduction or lack of CCN1 expression in MD cells in patients with CKD (Figure 6, A and B) suggests that CKD overrides the stimulatory effects of LS+ACEi observed in the normal healthy kidney (Figure 1 and Figure 4B).

The MD^geo^ cell line was another important tool to study MD cell signaling and secretome (Figure 4, A and B, and Supplemental Figure 4) and testing of the therapeutic potential of MD-derived biologicals (Figures 8 and 9). The use of either a specific, single (CCN1) or a mixture of all MD factors (conditioned medium) provided initial proof of concept of their therapeutic benefit (Figures 8 and 9). Compared with those before treatment, the improved albuminuria and GFR in response to treatment with LS-conditioned MD medium (and partially in response to treatment with CCN1) suggests functional regression of preexisting CKD (Figure 8A). The improvements in GS and interstitial fibrosis observed in animals treated with CCN1 or LS MD medium (Figure 9) are consistent with the known antifibrotic function of CCN1 (27, 54) and other MD-derived factors. It should be emphasized that the presently applied MD stimuli (LS+ACEi treatment, inducible MD-Wnt^gof^, MD biologicals) that resulted in improved kidney structure and function were all applied temporarily. However, sustained overactivation of the MD may become pathogenic, as seen, for example, in early diabetes (66). To determine whether the presently identified MD mechanisms are involved in the protective effects of SGLT2 inhibitors (66) or if their targeting provides additive or superior therapeutic benefit compared with existing treatments for kidney diseases requires further study.

## Methods

### Sex as a biological variable.

Our study examined male and female animals and patients, and similar findings are reported for both sexes.

### Animals.

Male and female 6- to 12-week-old C57BL6/J or BALB/c mice (The Jackson Laboratory) were used in all experiments. Some of the presently used transgenic mice were provided by academic investigators, such as the Cdh5 (PAC)-CreERT2 mice (by Ralf Adams, University of Münster, Münster, Germany; Cancer Research UK Scientist via Cancer Research Technology), the Ctnnb^lox(ex3)^ mice (by Makoto Mark Taketo, Kyoto University, Kyoto, Japan), and the Ren1d-Cre mice (Ariel Gomez, University of Virginia, Charlottesville, Virginia USA). Transgenic mouse models with the expression of various fluorescent reporter proteins and gene knockout strategies were generated by intercrossing Cre or Cre-ER^T2^ mice with flox mice and received a variety of treatments as listed and detailed in Supplemental Methods.

### Intravital imaging using MPM.

Surgical implantation of a dorsal abdominal imaging window above the left kidney was performed on Ng2-tdTomato and Cdh5-Confetti mice using aseptic surgical procedures as described recently (18). For repeated MPM imaging of the same mice with abdominal imaging window, animals underwent brief anesthesia sessions every 3–4 days using 1%–2% isoflurane and the SomnoSuite low-flow anesthesia system (Kent Scientific). For acute imaging experiments under continuous anesthesia (isoflurane 1–2% inhalant via nosecone), the left kidney was exteriorized through a flank incision. Mice were placed on the stage of the inverted microscope with the exposed kidney mounted in a coverslip-bottomed chamber bathed in normal saline and maintained as described previously (17, 19, 21). Alexa Fluor 594– or 680–conjugated albumin (Thermo Fisher) was administered i.v. by retro-orbital injections to label the circulating plasma (30 μL i.v. bolus from 10 μg/mL stock solution). The images were acquired using a Leica SP8 DIVE multiphoton confocal fluorescence imaging system with a Leica 25× water or 63× glycerine-immersion objective (numerical aperture [NA] 1.3) powered by a Chameleon Discovery laser (Coherent) and a DMI8 inverted microscope’s external Leica 4Tune spectral hybrid detectors (Leica Microsystems). Details regarding imaging settings, fluorescence quantification, and analysis are provided in Supplemental Methods.

### Unilateral IRI.

Unilateral clamping of the left renal pedicle was performed in anesthetized mice using isoflurane (1%–2% inhalant via nosecone), and body temperature was controlled at 37°C during surgery with a temperature-controlled operating table (SomnoSuite Low-Flow Anesthesia System, Kent Scientific). The left kidney was exteriorized through a 0.5 cm–long flank incision, and the left renal pedicle was dissected and clamped for 30 minutes of ischemia followed by the release of the clamp. The left kidney was inserted back into the retroperitoneum, and the wound was sutured. Animals that underwent only left kidney exteriorization but not left renal pedicle clamping (Sham operation) served as controls. At the end of 2 weeks of follow up, mice were euthanized, and tissues harvested for histological analysis.

### ADR nephropathy.

To establish the animal model of ADR-induced nephropathy, BALB/c mice at 8 weeks of age received a single i.v. injection of ADR (10.5 mg/kg, Sigma-Aldrich) via tail vein injection and were followed for a total of 6 weeks. At the 2-week disease peak, when GS pathology and albuminuria were stabilized (67), 4-week treatment of preexisting pathology was initiated in the following 5 groups by daily 200 μL i.p. injections: (a) control PBS, (b) low-dose CCN1 (angiogenic modulator, 5 ng/d), (c) high-dose CCN1 (1 mg/kg/d), (d) control DMEM-F12 cell culture medium, and (e) conditioned MD^geo^ cell culture medium. At the end of the 4-week treatment, mice were euthanized, and tissues harvested for histological analysis.

### GFR, albuminuria, and blood pressure measurements.

GFR measurements were performed using the MediBeacon Transdermal Mini GFR Measurement System (MediBeacon). Briefly, mice were anesthetized, and the MediBeacon sensor was placed on the depilated dorsal skin. Mice were injected retro-orbitally with the inulin analog exogenous GFR tracer FITC-conjugated sinistrin (FITC-S, 7.5 mg/100 g body weight, MediBeacon). The excretion kinetics of the FITC-S was measured for 90 minutes. GFR was then calculated based on the decay kinetics (half-life time) of FITC-S using MediBeacon Data Studio software (MediBeacon). Spot urine was collected from animals, and urine albumin was measured by using murine microalbuminuria ELISA kit (Albuwell M kits, Exocell). Urine creatinine was measured via microplate assay (The Creatinine Companion, Exocell), and ACR was calculated. Systolic blood pressure was measured by tail-cuff plethysmography (Visitech BP-2000, Visitech System Inc.) in trained animals as previously described (20).

### Tissue processing, immunofluorescence and immunoblotting, and histology.

Immunofluorescence detection of proteins was performed as described previously (20). Briefly, cryosections were cut at 25 μm and washed with 1× PBS. Paraffin tissue blocks were sectioned to 8 μm thick. For antigen retrieval, heat-induced epitope retrieval with sodium citrate buffer (pH 6.0) or Tris-EDTA (pH 9.0) was applied. To reduce nonspecific binding, sections were blocked with normal serum (1:20). Primary and secondary antibodies were applied sequentially overnight at 4°C and for 2 hours at room temperature. For immunoblotting of mouse cortical homogenates, manually dissected slices of kidney cortex were homogenized in a buffer containing 20 mM Tris·HCl, 1 mM EGTA pH 7.0, and a protease inhibitor cocktail (BD Bioscience). Protein (40 μg) was processed for immunoblotting as described previously (20). Primary antibodies and dilution details and other histology staining methods, including tissue clearing and their quantification, are provided in Supplemental Methods.

### MD cell isolation, immortalization, characterization, and conditioning.

MD-GFP cells were isolated as described before (12). Briefly, kidney cortex was isolated from freshly harvested mouse kidneys and digested using Hyaluronidase and Liberase enzyme combination (concentration, 2 mg/mL and 2.5 mg/mL, respectively, Sigma-Aldrich). After digestion, MD cells (GFP) and control cells (tdTomato) were isolated based on their genetic reporter expression by using FACS ARIAII cell sorter, and excitation wavelengths 488 and 633 nm in sterile conditions. The cells with the highest level of tdTomato expression were collected as controls, with high representation of distal tubule and collecting duct segments, podocytes, and vascular smooth muscle cells. The generation of the MD^geo^ cell line, characterization of NO and PGE2 synthesis and mass spectrometry analysis of secretome, and conditioning are provided in Supplemental Methods. MMDD1 cells (from J. Schnermann, NIH) were cultured as described before (23).

### RNA-Seq and bioinformatics.

Whole-transcriptome RNA-Seq was performed at the USC Norris Molecular Genomics Core using the Qiagen miRNeasy purification kit following manufacturer’s protocol for total RNA purification (Qiagen, 217004). Libraries were simultaneously prepared using Takara’s SMARTer Stranded Total-RNA Pico v2 library preparation kit following the manufacturer’s protocol (Takara, catalog 634412). Prepared libraries were sequenced on Illumina Nextseq500 over 75 cycles twice. Single-cell RNA-Seq was prepared using 10× Genomics 3′v3.1 (catalog 1000092) following manufacturer’s protocol as described before. Samples were parsed into single cells using 10× Genomics Chromium Controller, and libraries were simultaneously prepared. Prepared single-cell RNA-Seq libraries were sequenced on the Illumina Novaseq6000 platform at a read length of 28 × 90 and read depth of 100,000 reads/cell for 2,000–4,000 cells. RNA-Seq data were analyzed using the RNA-Seq workflow in Partek Flow software (V10.1.21., Partek Inc.) as described in Supplemental Methods.

### CCN1 assays.

The mouse CYR61 ELISA Kit (ab253223, Abcam) was used for the quantitative measurement of CCN1 protein in MD^geo^ cell culture media with or without conditioning according to the manufacturer’s instructions. Human MD cell CCN1, NOS1, and PTGS2 (COX2) protein expression was quantified based on immunohistochemical analysis of formalin-fixed, paraffin-embedded renal cortical tissues from unaffected regions of tumor nephrectomy specimens. Transcriptomic data analysis of *CCN1*, *NOS1*, and *PTGS2* was performed in human kidney biopsies from the ERCB. Human CCN1 ELISA assay (R&D Systems) was used to analyze urinary CCN1 in undiluted samples collected from controls (purchased from Bioreclamation IVT) and patients with clinically confirmed CKD (Clinical Phenotyping and Resource Biobank Core [C-PROBE] cohort based at University of Michigan, including patients with CKD stage I–V). Additional details of CCN1 analysis are provided in Supplemental Methods.

### Statistics.

Data represent average ± SEM and were analyzed using Student’s *t* tests (between 2 groups) or 2-(mixed-effect) or 1-way ANOVA (for multiple groups) with post-hoc comparison by Dunnett’s, Tukey’s, or Šidák’s tests as appropriate. *P* values of less than 0.05 were considered significant. Statistical analyses were performed using GraphPad Prism 9.0c.

### Study approval.

All animal protocols were approved by the Institutional Animal Care and Use Committee at the University of Southern California. The procurement and molecular analysis of renal biopsy, plasma, and urine samples have been approved by the Institutional Review Board at the University of Southern California (HS-15-00298 and HS-16-00378) and at the University of Michigan Health System (HUM00002468 and HUM00026609).

### Data availability.

Data from bulk RNA-based MD transcriptome analysis are available at the Gene Expression Omnibus (GEO) repository at the NCBI, with GEO accession GSE163576. For single-cell RNA-based MD transcriptome analysis, the GEO accession is GSE189954. All supporting values for this manuscript are provided in the Supporting Data Values file.

## Author contributions

GG and JPP designed the study, performed experiments, analyzed the imaging data, and wrote the manuscript. UNS, ARB, DD, WJ, SDS, AI, SD, ABC, JLB, HK, JYM, MMR, NA, LL, DB, AWJ, LM, BVZ, and MK performed experiments and made substantial contributions to acquire and analyze data. ISG provided clinical context and guidance. YC filtered and analyzed transcriptome data. All authors approved the final version of the manuscript.

## Supplementary Material

Supplemental data

Unedited blot and gel images

Supplemental table 1

Supplemental video 1

Supplemental video 2

Supporting data values

## Version 1 04/10/2024

In-Press Preview

## Version 2 06/03/2024

Electronic publication
