# Supplementary material for: Neuronally differentiated macula densa cells regulate tissue remodeling and regeneration in the kidney
Source: J Clin Invest. 2024 Apr 10;134(11):e174558. doi: 10.1172/JCI174558 (PMC11142747; doi:10.1172/JCI174558)
Supplement: Supplemental data [file jci-134-174558-s184.pdf]

Supplemental Materials

Neuronally differentiated macula densa cells regulate tissue remodeling and regeneration in the kidney

Georgina Gyarmati[1], Urvi Nikhil Shroff[1], Anne Riquier-Brison[1], Dorinne Desposito[1], Wenjun Ju[2], Sean D. Stocker[3], Audrey Izuhara[1], Sachin Deepak[1], Alejandra Becerra Calderon[1], James L Burford[1], Hiroyuki Kadoya[1], Ju-Young Moon[1], Yibu Chen[4], Markus M. Rinschen[5], Nariman Ahmadi[6], Lester Lau[7], Daniel Biemesderfer[8], Aaron W. James[9], Liliana Minichiello[10], Berislav V. Zlokovic[1], Inderbir S. Gill[6], Matthias Kretzler[2], János Peti-Peterdi[1*]

This file contains the following Supplemental Materials:

Supplemental Methods

Supplemental References

Supplemental Figures 1-6

Supplemental Methods

Animals

Transgenic mouse models with the expression of various fluorescent reporter proteins and gene knock-out strategies were generated by intercrossing Cre or Cre-ER^{T2} mice with flox mice as indicated in the table below.

Mouse Model	Cre/ Cre-ER^{T2}	Reporter	Knock-out model	Induction
Ng2-tdTomato	*Ng2* (1)	*tdTomato* (2)	-	tamoxifen via oral gavage once
Foxd1-tdTomato	*Foxd1* (3)	*tdTomato*	-	tamoxifen via oral gavage once at E18.5
Cdh5-Confetti	*Cdh5(*PAC) (4)	*(Gt(ROSA)26Sortm1(CAG-Brainbow2.1)Cle/J)* (5)	-	tamoxifen via oral gavage once
Ren1d-Confetti	*Ren1d* (6)	*(Gt(ROSA)26Sortm1(CAG-Brainbow2.1)Cle/J)*	-	-
MD-GFP	*Nos1* (7)	*Gt(ROSA)26Sortm4(ACTB-tdTomato,-EGFP)Luo* (8)	-	tamoxifen via oral gavage 3 times
MD-GFP-Wnt^{lof}	*Nos1*	*Gt(ROSA)26Sortm4(ACTB-tdTomato,-EGFP)Luo*	*Ctnnb* $^{lox(ex8-13)}$ (9)	tamoxifen via oral gavage 3 times
MD-GFP-Wnt^{gof}	*Nos1*	*Gt(ROSA)26Sortm4(ACTB-tdTomato,-EGFP)Luo*	*Ctnnb*$^{lox(ex3)}$ (10)	tamoxifen via oral gavage 3 times
Wnt-GFP	-	-	*TCF/Lef:H2B-GFP* (11)	
MD-Wnt^{lof} - Wnt-GFP	*Nos1*	-	*Ctnnb*$^{lox(ex8-13)}$ *TCF/Lef:H2B-GFP*	tamoxifen via oral gavage 3 times
MD-Wnt^{gof} - Wnt-GFP	*Nos1*	-	*Ctnnb*$^{lox(ex3)}$ *TCF/Lef:H2B-GFP*	tamoxifen via oral gavage 3 times
MD-GT	*Nos1*	B6;129S6-*Polr2aTn(pb-CAG-GCaMP5g,-tdTomato) Tvrd/J* (12)	-	tamoxifen via oral gavage 1 or 3 times
Sox2-GT	*Sox2* (13)	B6;129S6-*Polr2aTn(pb-CAG-GCaMP5g,-tdTomato) Tvrd/J*	-	-
MD-NGFR-KO	*Nos1*	B6;129S6-*Polr2aTn(pb-CAG-GCaMP5g,-tdTomato) Tvrd/J*	*Ngfrtm1.1Vk/BnapJ* (14)	tamoxifen via oral gavage 3 times
NGF-eGFP	-	-	*NGFpr-EGFP* (15)	

Tamoxifen was administered 75 mg/kg by oral gavage for a total of three times (every other day) for full induction, and one time for partial induction (16) resulting in cell specific expression of reporters and gene knock-out mouse models. A tamoxifen washout period of >2 weeks was always used. Some mice received low-salt or high-salt diet for 10 to 14 days (TD 90228 or TD 92012, respectively, Harlan Teklad, Madison, WI) with or without ACE inhibitor treatment using enalapril (150 mg/L via drinking water, E6888 purchased from Sigma Aldrich Solutions, MO) with or without selective COX2 inhibitor (SC58236, 6mg/L via drinking water, from Pfizer Inc. New York, NY), or selective NOS1 inhibitor 7-NI (20mg/kg ip daily injections, N778, Sigma Aldrich, St. Louis, MO); or furosemide (30mg/kg via ip injections daily, Chinoin, Budapest, Hungary). In addition, some mice received chronic nerve growth factor (NGF 0.03 mg/kg in 100 μL PBS sc., N8133, Millipore Sigma, Burlington, MA) treatment for 10 to 14 days, or acute intravenous bolus arginine vasopressin (AVP, 5μg/kg, V9879 Sigma Aldrich, St. Louis, MO), Furosemide (Furo, 1mg/kg, Sanofi-Aventis, Budapest, HU), or gastrin (1μg/kg, G1276 Sigma Aldrich, St. Louis, MO) injection. Some mice received low dose streptozotocin treatment (50ug/g body weight once a day for five consecutive days, S0130, Sigma Aldrich, St Louis, MO) to induce diabetes mellitus. Animals with blood glucose levels over 300mg/dL were considered diabetic.

Serial intravital imaging using multiphoton microscopy (MPM)

Images were acquired using a Leica SP8 DIVE multiphoton confocal fluorescence imaging system with a with a Leica 25× water or 63× glycerine-immersion objective (numerical aperture (NA) 1.3) powered by a Chameleon Discovery laser at either 860 nm or 970 nm (Coherent, Santa Clara, CA) and a DMI8 inverted microscope's external Leica 4Tune spectral hybrid detectors (emission at 460-480nm for CFP, 510-530 nm for eGFP and GCaMP5, 550-570 nm for YFP,

580-600 nm for tdTomato, 600-620 nm for RFP) (Leica Microsystems, Heidelberg, Germany). The potential toxicity of laser excitation and fluorescence to the cells was minimized by using a low laser power and high scan speeds to keep total laser exposure as minimal as possible. Image acquisition (12-bit, 512×512 pixel) consisted of only one z stack or time series per tissue volume (<2-10 min), which resulted in no apparent cell injury. For tissue volume and cell density analysis fluorescence images were collected in volume series (xyz, 1 s per frame) with the Leica LAS X imaging software and using the same instrument settings (laser power, offset, gain of all detector channels). Serial imaging of the same glomerulus in the same animal/kidney was performed once every 3 to 4 days for up 14 days after the first imaging session. Maximal projections from *Z*-stacks were used to count and compare tdTomato and Confetti^{+} cell number in the same area over time. In the Confetti mouse models clonal or unicolor tracing units were defined as numerous directly adjacent individual cells that featured the same Confetti color combination. All ten possible Confetti color combinations were observed as described before (22). The counting of tdTomato or Confetti^{+} cells and clones was facilitated by standardized image thresholding using ImageJ (NIH), Leica LAS X (Leica Microsystems Inc.), and cell-counting algorithms of Imaris 9.2 3D image visualization and analysis software (Bitplane USA) for intravital imaging *Z*-stacks. Clone frequency (the percentage of clones formed by 1, 2, 3-6, or >7 individual Confetti cells relative to all clones (100%)) was analyzed using mixed-effect analysis with two-way ANOVA. To study dynamic changes in intracellular calcium signaling in MD and other renal cell types, fluorescence images were collected in time series (xyt, 526 ms per frame) with the Leica LAS X imaging software and using the same instrument settings (laser power, offset, gain of both detector channels). The strong MD cell-specific tdTomato fluorescence signal (23) and high-

resolution MPM imaging allowed for easy identification of single MD cell bodies and processes. MPM imaging was always performed at the same time of day.

Quantification of GCaMP5 fluorescence intensity

An optical section including the vascular pole and the MD of a superficial glomerulus was selected, and time (xyt) series with 1 frame per 526 millisecond were recorded for 3 to 10 minutes to measure MD cell calcium dynamics. The strong, positive signal (GCaMP5/tdTomato fluorescence) and high-resolution MPM imaging allowed for easy identification of single MD, JG, EGM, IGM, EA and AA smooth muscle cell bodies. For the quantification of changes in mean GCaMP5 fluorescence intensity ROIs were drawn closely over the total cell body of single cells or over the entire MD plaque and the changes in GCaMP5 F/F_0 (green channel; fluorescence intensity expressed as a ratio relative to baseline) were measured after the experiment in the defined ROI using the Quantify package of LAS X software (version 3.6.0.20104; Leica-Microsystems). MD whole plaque data were smoothed using exponential 2nd order smoothing method (50 neighbors, GraphPad Prism). The frequency of MD calcium transients was measured based on the same defined ROIs using 3 to 10 minutes long time series recordings before and after manipulation. Maximum projection images of time series were generated (t=3min) to measure cumulative GCaMP5 fluorescence intensity over time.

Pearson's (*R*-based) connectivity analyses

Correlation analyses between the calcium signal time series for all MD cell pairs were performed with GraphPad Prism 9.0.1. as described (24). The correlation function R between all possible cell-pair combinations was assessed using Pearson's correlation. Data were displayed as heat-map

matrices, indicating individual cell-pair connections on each axis (minimum = −1; maximum = 1). $P < 0.001$ was deemed a statistically significant cell–cell connection. The coordinates of the imaged cells were used in the construction of connectivity line maps. Cell pairs ($R > 0.35$ and $P < 0.001$) were connected with a red straight line. MD cells with the most connections were labeled light red, MD cells without any apparent correlation to other MD cells were labeled light blue.

Tissue processing, immunofluorescence, RNAscope, and histology

Immunofluorescence detection of proteins was performed as described previously (28). Briefly, cryosections were cut at 25 µm, washed with 1x PBS. Paraffin tissue blocks were sectioned to 8 µm thick. For antigen retrieval, heat-induced epitope retrieval with Sodium Citrate buffer (pH 6.0) or Tris-EDTA (pH 9.0) was applied. To reduce non-specific binding, sections were blocked with normal serum (1:20). Primary and secondary antibodies were applied sequentially overnight at 4º C and 2 hours at room temperature. Primary antibodies and dilutions were as follows, anti-villin antibody (1:100, SC58897, Santa Cruz Biotechnology, Dallas, TX), anti-claudin1 antibody (1:100, SAB4503546, Sigma Aldrich, St. Louis, MO), anti-renin antibody (1:100, AF4277, R&D Systems, Minneapolis, MN), anti-alpha smooth muscle actin antibody (αSMA, 1:100, A2547, Sigma Aldrich, St. Louis, MO), anti- platelet-derived growth factor receptor beta antibody (PDGFRβ, 1:100, 3169S, Cell Signaling Technology, Danvers, MA), anti-podocin antibody (1:100, SC22298, Santa Cruz Biotechnology, Dallas, TX), anti-CCN1 antibody (1:100, SC13100, Santa Cruz Biotechnology, Dallas, TX), anti-growth differentiation factor 15 antibody (GDF15, 1:100, HPA011191, Sigma Aldrich, St. Louis, MO), anti-pappalysin2 antibody (PAPPA2, 1:100, PA5-21046, Invitrogen, Waltham, MA), anti-Wilms tumor protein antibody (WT1, 1:100, AB89901, Abcam, Cambridge, UK), anti-CD34 antibody, (1:100, AB8536, Abcam, Cambridge,

UK), anti-Meis2 antibody (1:100, HPA003256, Sigma Aldrich, St. Louis, MO), anti-tyrosine-hydroxylase antibody (TH, 1:100, AB152, Millipore Sigma, Burlington, MA), anti-calcitonin gene related peptide antibody (CGRP, 1:100, AB36001, Abcam Cambridge, UK), anti-synaptophysin antibody (SYP, 1:100, PA5-27286, Thermo Fisher, Waltham, MA), anti-nerve growth factor receptor antibody (p75NTR, 1:100, AB227509, Abcam Cambridge, UK), anti-tropomyosin kinase A/B antibody (TRKA/B, 1:100, A7H6R, Cell Signaling Technology Danvers, MA), anti-cyclooxygenase 2 (COX2) antibody (1:100, 12282S, Cell Signaling Technology, Danvers, MA,), anti-Na-K-2Cl cotransporter antibody (NKCC2, 1:100, Developmental Studies Hybridoma Bank, created by the NICHD of the NIH and maintained at The University of Iowa, Department of Biology, Iowa City, IA 52242), anti-phospho-Tau antibody ($pTau^{S199}$, 1:100, 44-734G, Waltham, MA), anti-GFP antibody (GFP, 1:200, A10262, Thermo Fisher Scientific, Waltham, MA), anti-kidney injury molecule 1 antibody (KIM1, 1:100, NBP1-76701, Novus Biologicals, Centennial, CO), anti-plasmalemmal vesicle associate protein (PLVAP, 1:100, MECA-32, Bio-Rad Laboratories, Hercules, CA). Alexa Fluor 488, 594, and 647-conjugated secondary antibodies were purchased from Invitrogen. Slides were mounted by using DAPI-containing mounting media (VectaShield, Vector Laboratories Inc., Burlingame, CA). The expression of Axin2 was quantified using RNAscope™ Probe Mm-Axin2-C3 (400331-C3, Advanced Cell Diagnostics, Newark, CA) according to the manufacturer's instructions. Sections were examined with Leica TCS SP8 (Leica Microsystems, Wetzlar, Germany) confocal/multiphoton laser scanning microscope systems as described previously (16). Imaging software (Image J, National Institutes of Health and the Laboratory for Optical and Computational Instrumentation (LOCI, University of Wisconsin) was used to calculate signal density. The percent area of colocalization was calculated to determine the changes in the

proportion of $NG2^{+}$ cells in each cell type, as well as the proportional distribution of $NG2^{+}$cell types.

For the evaluation of glomerulosclerosis, histological analysis of periodic acid-Schiff (PAS) staining was performed on mouse kidney sections using PAS Stain Kit (24200-1, Polysciences, Warrington, PA). For the assessment of tubulointerstitial fibrosis, histological analysis of Picrosirius red (PSR) staining was performed on mouse kidney sections using Sirius Red F3B (Sigma-Aldrich, St. Louis, MS). Images were visualized at 25× magnification using Leica TCS SP8 (Leica Microsystems, Wetzlar, Germany). Quantification of glomerulosclerosis and tubulointerstitial fibrosis was evaluated blindly by measuring the density of PAS or PSR staining on histological sections (n=5 glomeruli/tissue areas averaged per animal) using Image J as described before (29). Podocyte (p57+ cell) number was calculated in identical tissue volumes using projection images.

Antibodies that were used in immunostaining and blotting performed in our laboratory were validated using specific blocking peptides if these were available. Some of the human kidney immunohistochemistry images were obtained from the Human Protein Atlas (http://www.proteinatlas.org) that were obtained with antibodies that have not been fully validated in the current studies (30).

Tissue CLARITY

Three-dimensional imaging was performed as previously described (31) by carrying out whole-mount immunofluorescence stains on slices of MD-GFP WT, Wnt^{gof}, and Wnt^{lof} mouse kidneys. Slices were fixed in 4% formaldehyde in 1x phosphate buffer saline (PBS) at room temperature for 45 min, washed in 1XPBS, blocked in 1xPBS with 0.1% TritonX100 and 2% SEA Block

(ThermoFisher Scientific) for 1 hour, and sequentially incubated in primary and secondary antibodies over 2 days. Primary antibodies and dilutions were as follows: anti-Wilms tumor protein antibody (WT1, 1:100, AB89901, Abcam, Cambridge, UK), anti-Meis2 antibody (1:100, HPA003256, Sigma Aldrich, St. Louis, MO), tyrosine-hydroxylase (AB152, MilliporeSigma, 1:100), GFP (ThermoFisher, 1:200). Primary and secondary antibodies were diluted in the blocking solution. To clear tissue slices, the slices were dehydrated in methanol via increasing concentrations 50%, 75%, 100%, diluted in PBS - each for 1hr - and subsequently submerged in a 50:50 benzyl benzoate/benzyl alcohol (BABB): methanol solution, followed by 100% BABB. High resolution imaging of MD plaques and the adjacent glomeruli was performed on a Leica SP8 multiphoton microscope using a 63X glycerol immersion objective.

RNA sequencing and bioinformatics

Whole-transcriptome RNAseq was performed at the USC Norris Molecular Genomics Core as described before (32). Cells were extracted using Qiagen miRNeasy purification kit following manufacturer's protocol for total RNA purification (Qiagen cat#217004). Libraries were simultaneously prepared using Takara's SMARTer Stranded Total-RNA Pico v2 library preparation kit following manufacturer's protocol (Takara cat#634412). Prepared libraries were sequenced on Illumina Nextseq500 at 2x75cycles.

RNA-seq data was analyzed using the RNA-seq workflow in Partek Flow software (V10.1.21. Partek Inc., St. Louis, MO, USA). Briefly, the raw sequencing reads were first trimmed based on the quality score (Phred QC>=20, min read length=25 nt) before mapped to mouse genome build mm10 using Star 2.61 (33) with default parameter settings and Gencode M21 mouse transcriptome annotation (34) as guidance. Gencode M21 was then used to quantify the aligned reads to genes

using Partek E/M method. Finally, gene level read counts in all samples were normalized using Upper Quartile normalization (35) and subjected to differential expression analysis using Partek Gene Specific Analysis method (genes with fewer than 10 aligned reads in any sample among a data set were excluded). The differentially expressed gene (DEG) lists were generated using the cutoff of FDR<0.05 and fold changes greater than 2.0 either direction. Z-scores based on the normalized gene counts from the top MD cell enriched genes in 5 categories were used to generate the heatmap in GraphPad Prism 9.0.1 (San Diego, California). Pathway analysis, graphical summary, biological and disease functions of IPA were used to analyze transcriptome data.

Single cell RNA sequencing was prepared using 10x Genomics 3' v3.1 (cat# 1000092) following manufacturer's protocol as described before. Samples were parsed into single cells using 10x Genomics Chromium Controller and libraries were simultaneously prepared. Prepared single cell RNA sequencing libraries were sequenced on the Illumina Novaseq6000 platform at a read length of 28x90 and read depth of 100,000 reads/cell for 2000-4000 cells. scRNAseq data was analyzed using the scRNAseq workflow by Partek Flow. Briefly, the raw sequencing reads with adaptors trimmed were mapped mm10 genome using Star 2.6.1 and quantified using Gencode M25 annotation to generate gene level counts. The gene counts were subjected to QA/QC and the low-quality cells were filtered using the following criteria: (1) contained less than 300 or more than 8000 detected genes, (2) mitochondrial counts higher than 15% of total counts. The counts were normalized using the Partek Flow recommended method (divided by 1 million, Add:1 and log2). Dimension reduction was carried out using PCA, followed by Graph-based Clustering with default settings and UMAP visualization (36). Cell populations were determined by expression of relevant biomarkers. A 5-fold thresholding in *Nos1* and *Pappa2* expression was applied to filter out

potential non-MD cell contamination. *Nos1/Pappa2* expression filtered cells went through PCA, Graph-based clustering and UMAP visualization.

Generation of the MD^{geo} cell line

Freshly isolated MD cells were plated at a density of $0.5^{x}10^{5}$ cells/well in a 24-well plate and primary cultured at 37 °C 5% CO_2 in Dulbecco's Modified Eagle Medium: Nutrient Mixture F-12 (DMEM-F12, Gibco, Thermo Fisher Scientific) supplemented with 10% Fetal Bovine Serum (FBS, Thermo Fisher Scientific), 1% Penicillin-Streptomycin (P/S 10,000 U/mL, Thermo Fisher Scientific) and 0.0005% of Dexamethasone. At 80% confluence, cells were infected using Lenti-SV40 (ts58 temperature sensitive mutant, 10^{6} IU/mL, LV629, Applied Biological Materials) Lentivirus to achieve temperature sensitive immortalization, proliferation of MD cells at 33 °C and differentiation of MD cells at 37 °C according to manufacturer's instructions. Briefly, cells were infected two times 8 hours apart using MOI:3 to achieve optimal viral density and incubated for 24 hours at 37°C, 5% CO_2 in the presence of Polybrene (5ug/mL). Lentiviral vector was diluted with complete MD cell culture media to avoid cytotoxicity. After 24 hours incubation cells were cultured in complete MD cell culture media supplemented with IFN-Gamma (0.01ug/mL) and nerve growth factor (NGF, 0.1 ug/mL; N8133, Millipore Sigma) at 33°C for proliferation. IFN-Gamma concentration was decreased gradually after 1 week. Importantly, NGF supplementation was crucial for MD cell survival. Cells were subcultured after 48 hours. Cell proliferation rate was calculated based on cell counting using INCYTO C-Chip hemocytometer (Burker Turk, Thomas Scientific, Swedesboro, NJ). For differentiation, cells were incubated at 37°C, 5% CO2 for 14 days in complete MD cell culture media supplemented with NGF (0.1ug/mL; N8133, Millipore Sigma). All experiments were completed between 10th and 15th cell passage.

NO assay

To assess nitric oxide (NO) synthesis in MD^{geo} cells NO sensitive Diaminofluorescein-FM diacetate (D1946, DAF-FM DA, Millipore Sigma, Burlington, MA) was used as described before (37). Briefly, fully differentiated MD^{geo} cells were loaded with DAF-FM DA (10µg/mL for 10 minutes) either in the presence or absence of the selective inhibitor of neuronal nitric oxide synthase (7-Nitroindazole (7-NI, 50 µM, N7778, Millipore Sigma) or Nω-propyl-l-arginine (NPA, 300 mM, SML2341, Millipore Sigma)). NO synthesis was measured based on the increase in the fluorescence intensity of DAF-FM DA (F/F_0 green channel; fluorescence intensity expressed as a ratio relative to baseline) over time. Fluorescence emission was detected at baseline, and after 30, 60, and 90 minutes at 515 +/- 15 nm emission wavelength in response to 495 nm excitation using Leica SP8 DIVE multiphoton confocal fluorescence imaging system. Changes in DAF-FM DA F/F_0 were measured after the experiment using the Quantify package of LAS X software (3.6.0.20104; Leica-Microsystems)

PGE2 biosensor technique

PGE2 biosensor cells were used as described before (38). In brief, PGE2 biosensor cells were specifically engineered human embryonic kidney cells (HEK 293 cells) to express the calcium-coupled PGE2 receptor EP1, leading to a calcium response upon PGE2 binding. HEK-EP1 cells were loaded with Fluo-4 and Fura Red (1 µM, for 10 min) and positioned next to the fully differentiated MD^{geo} cells in culture. MD^{geo} cell PGE2 production was measured based on the biosensor cell intracellular Ca^{2+} concentration signal, which was detected by increases in Fluo-4/Fura Red (F/F_0) fluorescence intensity ratio over time. Fluorescence emission was detected every

526 ms using a Leica SP8 DIVE multiphoton confocal fluorescence imaging system as described above (emission at 460-520 nm for Fluo-4 and at 580-640 nm for Fura Red) (Leica Microsystems, Heidelberg, Germany).

Generation of conditioned MD^{geo} cell culture media

The newly established immortalized macula densa cell line (MD^{geo}) was cultured and differentiated as described above. After full differentiation, MD^{geo} cells were washed in PBS to remove FBS and NGF and were physiologically activated by temporary exposure to low-salt conditions (low salt DMEM-F12 medium) (39) for 6 hours every other day (3 times). Cell culture media was collected in sterile conditions and stored at -80 C until treatment.

Western Blot

For immunoblotting of mouse cortical homogenates, manually dissected slices of kidney cortex were homogenized in a buffer containing 20 mM Tris·HCl, 1 mM EGTA pH 7.0, and a protease inhibitor cocktail (BD Bioscience, San Jose, CA). Protein (40 μg) was processed for immunoblotting as described previously (28). Primary antibodies and dilutions were as follows: anti-CCN1 (1:500, SC13100, Santa Cruz Biotechnology, Dallas, TX), anti-SEMA3C (1:500, MAB1728, R & D Systems, Minneapolis, MN), anti-CCN3 (1:1000, ab137677, Abcam, Cambridge, UK), anti-CXCL14 (1:1000, NBP1-31398, Novus Biologicals, Centennial, CO), anti-p75NTR antibody (1:1000, ab227509, Abcam, Cambridge, UK), anti-COX2 antibody (1:1000, 12282S, Cell Signaling Technology, Danvers, MA), anti-NKCC2 cotransporter antibody (1:1000, Developmental Studies Hybridoma Bank, created by the NICHD of the NIH and maintained at The University of Iowa, Department of Biology, Iowa City, IA 52242), anti-neuronal nitric oxide

synthase antibody (NOS1, 1:1000, SC648, Santa Cruz Biotechnology, Dallas, TX) anti-phospho and total extracellular signal-regulated kinase antibody (p/total ERK1-2, 1:1000, CST4396, CST4696, Cell Signaling Technology, Danvers, MA), anti-phospho and total protein kinase B antibody (AKT, 1:1000, CST2920, CST4060, Cell Signaling Technology, Danvers, MA), anti-phospho and total IkB kinase antibody (p/total IkB, 1:1000, 1:500, CST9246S, CST924, Cell Signaling Technology, Danvers, MA), anti-phospho and total panTrk (1:1000, 4619S, 92991S, Cell Signaling Technology, Danvers, MA), anti-renal outer medullary potassium channel antibody (ROMK, 1:2000, APC-001, Alomone Labs, Jerusalem, Israel), anti-phospho and total p38 mitogen-activated protein kinase antibody (p/total p38, 1:1000, 9211, 9212, Cell Signaling Technology, Danvers, MA). After incubation, blots were incubated with secondary antibodies (1:5000; LI-COR Biosciences) and then visualized with Odyssey Infrared Imaging System (LI-COR Biosciences). Protein staining of the gel (Coomassie staining) was performed and analyzed to confirm equal loading as described previously (40).

Mass spectrometry analysis of the composition of MD^{geo} cell culture media

Cell-free MD^{geo} conditioned media were snap frozen and stored at −80°C. Proteins were precipitated using acetone. Proteins were digested and prepared using the SP3 protocol, with modifications, as previously described, using trypsin as a protease (41). Tryptic peptides were analyzed using a nanoscale liquid chromatography–tandem mass spectrometry hardware setup, consisting of a nanoflow LC (flow, 200 nl/min) coupled to an Orbitrap QExactive Plus tandem mass spectrometer. The peptides were separated using a gradient for reverse-phase separation, consisting of buffer A and buffer B, with ascending concentrations of buffer B (80% acetonitrile,

0.1% formic acid) over buffer A (0.1% formic acid). The peptides were separated using a 1-hour gradient.

Protein raw files were searched using MaxQuant and the LFQ algorithm (42, 43) with searches against a UniProt mouse proteome reference database released in January 2018. Search criteria were alkylation on cysteines as a fixed modification, and amino-terminal acetylation and methionine oxidation as variable modifications. Default criteria were used, meaning that PSM, peptide, and protein false discovery rates (FDRs) were set at 0.01. The LFQ algorithm was enabled, and "match between run" was enabled. The data were analyzed using Perseus version 1.5.5.3, with filtering for the embedded annotations as contaminant, reverse, or proteins identified by site only.

CCN1 protein analysis

Human MD cell CCN1, NOS1, and PTGS2 (COX2) protein expression was quantified based on immunohistochemical analysis using anonymized adult (32-87 years old) formalin-fixed paraffin-embedded renal cortical tissues and reagents as above. Samples were obtained from unaffected regions of tumor nephrectomy specimens based on protocols HS-15-00298 and HS-16-00378 approved by the Institutional Review Board, Keck School of Medicine of the University of Southern California. Non-diabetic nephrectomy patients with either normal kidney function or CKD due to hypertensive nephropathy (eGFR <50 mL/min/1.73m^2) were included. Basic patient history data regarding kidney function (eGFR) and comorbidities, such as hypertension, and medications were available.

The quantification method of CCN1, NOS1, and PTGS2 expression included the selection of 10 glomeruli in each sample in which the entire MD longitudinal section (glomerular mid-sections containing both vascular and urinary poles) was available. The number of CCN1$^+$, NOS1$^+$, and

$PTGS2^+$ individual MD cells per MD plaque were counted in a blinded fashion and the average of 5 MDs/glomeruli were used per kidney sample, n=6 patients (3 male and 3 female) each in control and CKD groups.

Gene expression analysis

Transcriptomic data analysis of *CCN1, NOS1*, and *PTGS2* was performed in human kidney biopsies from the European Renal cDNA Biobank (ERCB)(44). For tissue transcriptome analysis, gene expression profiles from 174 consecutive biopsies of patients with various CKD etiologies were compared with those from thirty-two living donor transplant biopsies obtained at the time of transplantation. Only patients aged 18 years or older were included in this study.

Transcriptome analysis was performed on microdissected tubulointerstitial components of human renal biopsies prospectively procured for molecular analysis, using Affymetrix GeneChip and TaqMan Low Density Arrays as previously published (45). Normalized expression data were log2-transformed and batch-corrected. FDR was applied to account for multiple testing.

Urinary CCN1 analysis

Human CCN1 ELISA assay (R&D systems, Minneapolis, MN, USA) was used to analyze urinary CCN1 in undiluted samples collected from control (n=11) and patients with clinically confirmed CKD (n=29). Control samples were purchased from Bioreclamation IVT (New York, NY, USA). CKD samples were from the Clinical Phenotyping and Resource Biobank Core (C-PROBE) cohort based at University of Michigan that includes patients with CKD stage I-V. Spearman correlation analysis was performed between urinary CCN1/Creatinine levels and eGFR as described earlier (46).

Supplemental References

1. Zhu X, et al. Age-dependent fate and lineage restriction of single NG2 cells. *Development.* 2011;138(4):745-53.
2. Madisen L, et al. A robust and high-throughput Cre reporting and characterization system for the whole mouse brain. *Nat Neurosci.* 2010;13(1):133-40.
3. Humphreys BD, et al. Fate tracing reveals the pericyte and not epithelial origin of myofibroblasts in kidney fibrosis. *Am J Pathol.* 2010;176(1):85-97.
4. Wang Y, et al. Ephrin-B2 controls VEGF-induced angiogenesis and lymphangiogenesis. *Nature.* 2010;465(7297):483-6.
5. Snippert HJ, et al. Intestinal crypt homeostasis results from neutral competition between symmetrically dividing Lgr5 stem cells. *Cell.* 2010;143(1):134-44.
6. Sequeira López ML, et al. Renin cells are precursors for multiple cell types that switch to the renin phenotype when homeostasis is threatened. *Dev Cell.* 2004;6(5):719-28.
7. Taniguchi H, et al. A resource of Cre driver lines for genetic targeting of GABAergic neurons in cerebral cortex. *Neuron.* 2011;71(6):995-1013.
8. Muzumdar MD, et al. A global double-fluorescent Cre reporter mouse. *Genesis.* 2007;45(9):593-605.
9. Brault V, et al. Inactivation of the beta-catenin gene by Wnt1-Cre-mediated deletion results in dramatic brain malformation and failure of craniofacial development. *Development.* 2001;128(8):1253-64.
10. Harada N, et al. Intestinal polyposis in mice with a dominant stable mutation of the beta-catenin gene. *Embo j.* 1999;18(21):5931-42.
11. Ferrer-Vaquer A, et al. A sensitive and bright single-cell resolution live imaging reporter of Wnt/ß-catenin signaling in the mouse. *BMC Dev Biol.* 2010;10(121.
12. Gee JM, et al. Imaging activity in neurons and glia with a Polr2a-based and cre-dependent GCaMP5G-IRES-tdTomato reporter mouse. *Neuron.* 2014;83(5):1058-72.
13. Hayashi S, et al. Efficient gene modulation in mouse epiblast using a Sox2Cre transgenic mouse strain. *Mech Dev.* 2002;119 Suppl 1(S97-s101.
14. Bogenmann E, et al. Generation of mice with a conditional allele for the p75(NTR) neurotrophin receptor gene. *Genesis.* 2011;49(11):862-9.
15. Kawaja MD, et al. Nerve growth factor promoter activity revealed in mice expressing enhanced green fluorescent protein. *J Comp Neurol.* 2011;519(13):2522-45.
16. Gyarmati G, et al. A new view of macula densa cell microanatomy. *Am J Physiol Renal Physiol.* 2021;320(3):F492-f504.
17. Sun YB, et al. Glomerular endothelial cell injury and damage precedes that of podocytes in adriamycin-induced nephropathy. *PLoS One.* 2013;8(1):e55027.
18. Ritsma L, et al. Surgical implantation of an abdominal imaging window for intravital microscopy. *Nat Protoc.* 2013;8(3):583-94.
19. Kaverina NV, et al. Tracking the stochastic fate of cells of the renin lineage after podocyte depletion using multicolor reporters and intravital imaging. *PLoS One.* 2017;12(3):e0173891.
20. Hackl MJ, et al. Tracking the fate of glomerular epithelial cells in vivo using serial multiphoton imaging in new mouse models with fluorescent lineage tags. *Nat Med.* 2013;19(12):1661-6.

21. Kang JJ, et al. Quantitative imaging of basic functions in renal (patho)physiology. *Am J Physiol Renal Physiol.* 2006;291(2):F495-502.
22. Desposito D, et al. Serial intravital imaging captures dynamic and functional endothelial remodeling with single-cell resolution. *JCI Insight.* 2021;6(10).
23. Shroff UN, et al. A new view of macula densa cell protein synthesis. *Am J Physiol Renal Physiol.* 2021.
24. Salem V, et al. Leader β-cells coordinate Ca(2+) dynamics across pancreatic islets in vivo. *Nat Metab.* 2019;1(6):615-29.
25. DeLalio LJ, and Stocker SD. Impact of anesthesia, sex, and circadian cycle on renal afferent nerve sensitivity. *Am J Physiol Heart Circ Physiol.* 2021;320(1):H117-h32.
26. DeLalio LJ, and Stocker SD. Impact of anesthesia and sex on sympathetic efferent and hemodynamic responses to renal chemo- and mechanosensitive stimuli. *J Neurophysiol.* 2021;126(2):668-79.
27. Scarfe L, et al. Transdermal Measurement of Glomerular Filtration Rate in Mice. *J Vis Exp.* 2018140).
28. Riquier-Brison ADM, et al. The macula densa prorenin receptor is essential in renin release and blood pressure control. *Am J Physiol Renal Physiol.* 2018;315(3):F521-f34.
29. Ranjit S, et al. Label-free fluorescence lifetime and second harmonic generation imaging microscopy improves quantification of experimental renal fibrosis. *Kidney Int.* 2016;90(5):1123-8.
30. Uhlén M, et al. Proteomics. Tissue-based map of the human proteome. *Science.* 2015;347(6220):1260419.
31. Lindström NO, et al. Conserved and Divergent Features of Human and Mouse Kidney Organogenesis. *J Am Soc Nephrol.* 2018;29(3):785-805.
32. Gyarmati G, et al. Neuron-like function of the nephron central command. *bioRxiv.* 2021:2021.12.06.471478.
33. Dobin A, et al. STAR: ultrafast universal RNA-seq aligner. *Bioinformatics.* 2013;29(1):15-21.
34. Mudge JM, and Harrow J. Creating reference gene annotation for the mouse C57BL6/J genome assembly. *Mamm Genome.* 2015;26(9-10):366-78.
35. Bullard JH, et al. Evaluation of statistical methods for normalization and differential expression in mRNA-Seq experiments. *BMC Bioinformatics.* 2010;11(94.
36. McInnes L, et al. UMAP: Uniform Manifold Approximation and Projection. *Journal of Open Source Software.* 2018;3(29):861.
37. Kovács G, et al. Neuronal nitric oxide synthase: its role and regulation in macula densa cells. *J Am Soc Nephrol.* 2003;14(10):2475-83.
38. Peti-Peterdi J, et al. Luminal NaCl delivery regulates basolateral PGE2 release from macula densa cells. *J Clin Invest.* 2003;112(1):76-82.
39. Yang T, et al. Low chloride stimulation of prostaglandin E2 release and cyclooxygenase-2 expression in a mouse macula densa cell line. *J Biol Chem.* 2000;275(48):37922-9.
40. McDonough AA, et al. Considerations when quantitating protein abundance by immunoblot. *Am J Physiol Cell Physiol.* 2015;308(6):C426-33.
41. Rinschen MM. Single glomerular proteomics: A novel tool for translational glomerular cell biology. *Methods Cell Biol.* 2019;154(1-14.

42. Cox J, and Mann M. MaxQuant enables high peptide identification rates, individualized p.p.b.-range mass accuracies and proteome-wide protein quantification. *Nat Biotechnol.* 2008;26(12):1367-72.
43. Cox J, et al. Accurate proteome-wide label-free quantification by delayed normalization and maximal peptide ratio extraction, termed MaxLFQ. *Mol Cell Proteomics.* 2014;13(9):2513-26.
44. Yasuda Y, et al. Gene expression profiling analysis in nephrology: towards molecular definition of renal disease. *Clin Exp Nephrol.* 2006;10(2):91-8.
45. Schmid H, et al. Modular activation of nuclear factor-kappaB transcriptional programs in human diabetic nephropathy. *Diabetes.* 2006;55(11):2993-3003.
46. Ju W, et al. Tissue transcriptome-driven identification of epidermal growth factor as a chronic kidney disease biomarker. *Sci Transl Med.* 2015;7(316):316ra193.

Supplemental Figures

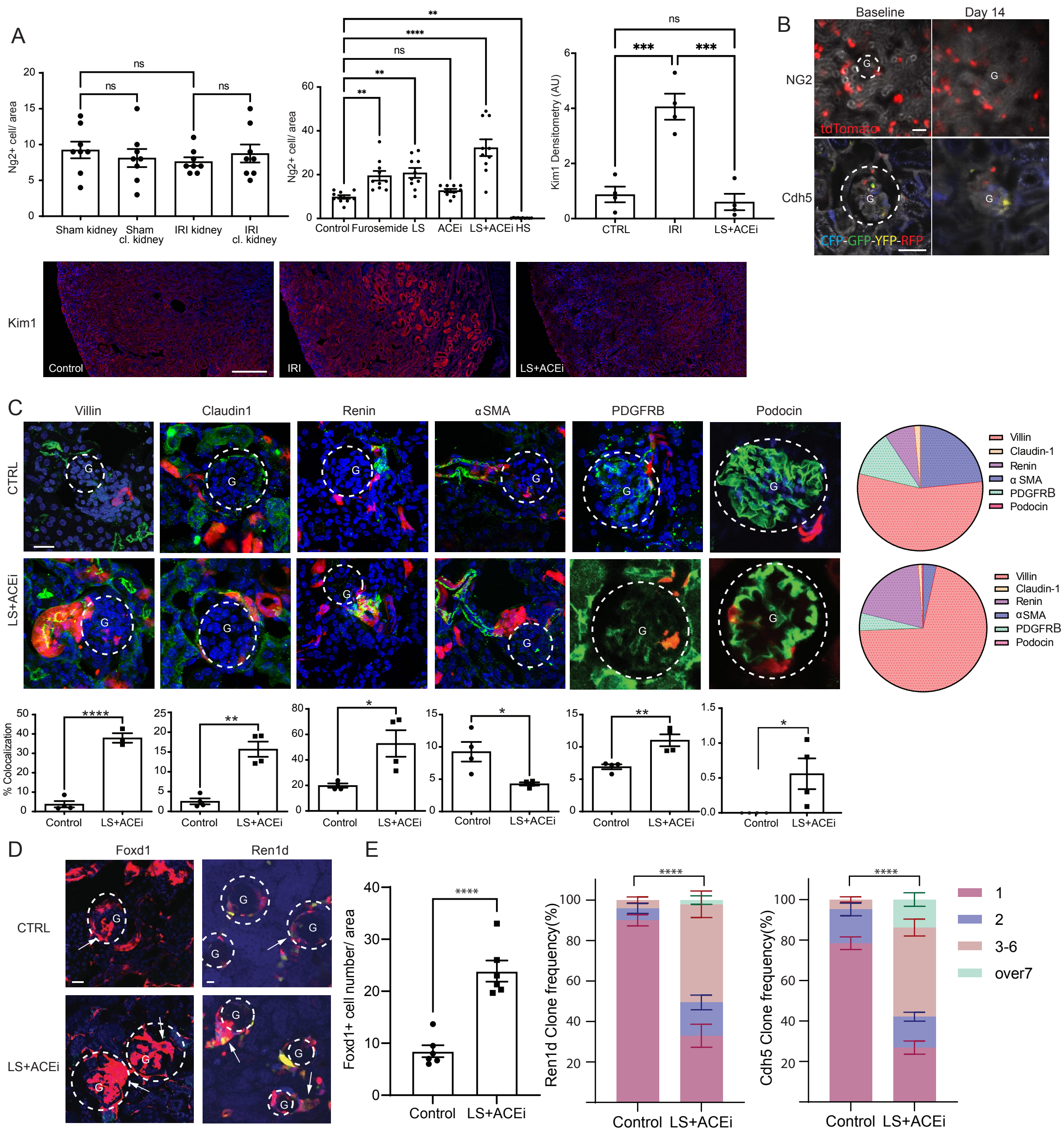

Supplemental Figure 1. Additional features of mesenchymal and endothelial precursor cell-mediated endogenous kidney tissue remodeling.

(A) The effects of ischemia-reperfusion injury (IRI) or sham surgery in the ipsilateral or contralateral (cl) kidney (left) or treatment with furosemide, or low-salt (LS) diet or ACEi alone or in combination (LS+ACEi), or high-salt diet (center) on $Ng2^+$ cell number per glomerular area. Kim1 immunolabeling on fixed kidney tissue sections confirmed the presence of tissue injury in IRI, but not in control or LS+ACEi conditions (right and bottom), n=4-10 (average of 10 glomeruli/animal). Bar: 250 µm.

(B) In vivo MPM images of time control experiments (same glomeruli (G, dashed white circle) at baseline and at 14 days of control normal-salt diet) in Ng2-tdTomato (mesenchymal cells in red) and Cdh5-Confetti (endothelial cells in Confetti multicolor) mice. Bar: 50 µm.

(C) Fate tracking of mesenchymal progenitor ($Ng2^+$, red) cells in the renal cortex in timed control (top row) or after treatment with LS+ACEi for two weeks (center row). Native $Ng2^+$ cell tdTomato (red) fluorescence images with immunofluorescence co-localization of cell differentiation markers (green) for proximal tubule (villin), parietal epithelial (claudin-1), juxtaglomerular renin (renin), vascular smooth muscle (αSMA), and mesangial cells (PDGFRβ), and podocytes (podocin) in timed control or after LS+ACEi treatment (left). The relative distribution of the six differentiated cell types within the $Ng2^+$ cell population is quantified in pie charts (right). The ratio of $Ng2^+$ cells within each of the six differentiated cell types (% co-localization of $double^+$/green cells) is shown below each cell type (bottom row), n=4 (average of 10 areas/animal). Note that the percentage of $αSMA^+$ and $PDGFRβ^+$ cells within the Ng2 lineage decreased, while all other cell types increased in response to LS+ACEi treatment. Bar: 50 µm.

(D) Native fluorescence images of tdTomato (red) in $Foxd1^+$ cells (left) or Confetti (CFP/GFP/YFP/RFP multicolor) in $Ren1d^+$ cells (right) in timed control or after LS+ACEi treatment in Foxd1-tdTomato or Ren1d-Confetti mouse kidney sections. Glomerular vascular pole

areas under the MD cell base are indicated by arrows in multiple nephrons. Nuclei are labeled blue with DAPI. G: glomerulus. Bar: 50 μm.

(E) Quantification of Foxd1$^+$ cell number and Ren1d$^+$ or Cdh5$^+$ clone frequency per glomerular area in timed control or after LS+ACEi treatment, n=4-6 (average of 3-10 glomeruli/animal). Data represent mean ± SEM, ns: not significant, *p<0.05, **p<0.01, ***p<0.001, ****p<0.0001 t-test (C, E) or ANOVA followed by Sidak's test (A, left panel), or Dunnett's test (A, center panel), or Tukey's test (A, right panel).

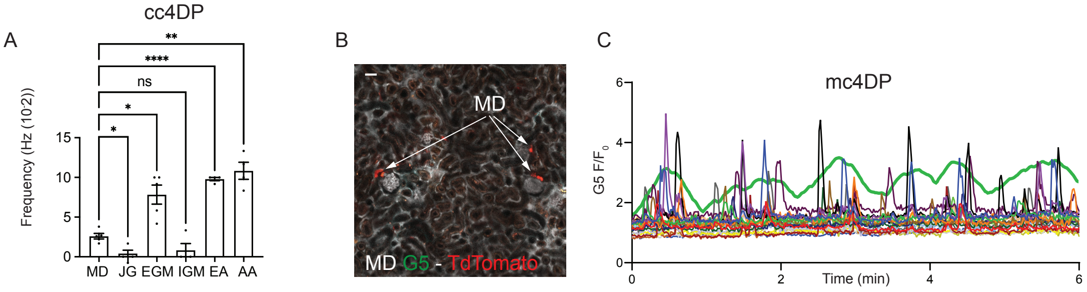

Supplemental Figure 2. Additional features of Sox2-GT and MD-GT mice.

(A) Intravital MPM imaging of intracellular calcium in glomerular vascular pole cell types in cc4DP mode. Comparison of the frequencies of Ca^{2+} transients in macula densa (MD), juxtaglomerular (JG) renin, extra (EGM) and intra-glomerular (IGM) mesangial cells, and afferent (AA) and efferent arteriole (EA) vascular smooth muscle cells, n=4-5 (average of 4-5 cells/animal).

(B) Tile-scan overview image of a renal cortical surface area of MD-GT mice (mc4DP and sc4DP modes) demonstrating the expression of GCaMP5 (G5, green) and tdTomato (red) reporters exclusively in MD cells (arrows) at the vascular pole of glomeruli. Alexa Fluor 680-conjugated bovine serum albumin was injected iv to label the circulating plasma (greyscale). Bar: 50 μm.

(C) Overlay of Ca^{2+} recordings of all 21 individual MD cells (each cell in a different color) shown in Figure 2C (mc4DP mode). Note the clustering of single-cell Ca^{2+} transients and their periodic oscillations over time (curve fitting in green).

Data represent mean ± SEM, ns: not significant, *p<0.05, **p<0.01, ***p<0.001, ****p<0.0001, ANOVA followed by Dunnett's test.

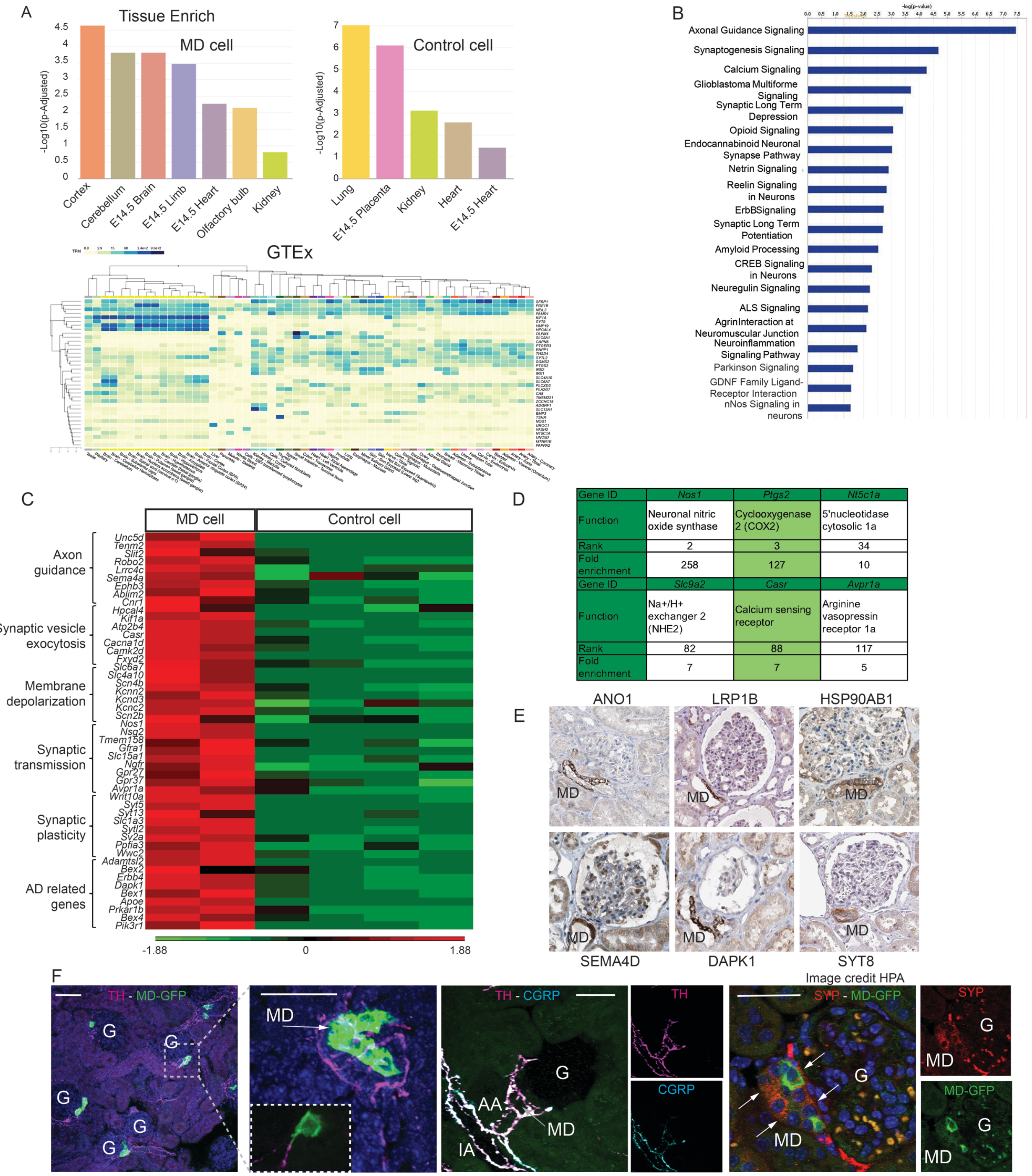

Supplemental Figure 3. MD cell gene profile featuring neuronal differentiation.

(A) Tissue specificity analysis of MD (left) and control cell (right) transcriptome using bulk RNA seq. Bar chart showing the enrichment (-Log10(P-Adjusted)) of the top 50 highest expressed MD

cell or control cell-specific genes in various tissues using TissueEnrich and the Mouse ENCODE dataset for comparison. Heat map of the expression of the same top MD cell gene set in various tissues using GTEx Multi Gene Query (bottom).

(B) Transcriptome analysis of the most significant MD-specific canonical pathways based on IPA analysis.

(C) Heat map of MD (red) vs. control renal cell (green) expression of the top 50 neuronal differentiation-specific MD cell enriched genes in 6 GO term categories as indicated based on Partek Flow analysis (n=2 MD and n=4 control).

(D) Examples of established MD-specific genes that are highly expressed in MD vs. control cells (extract from Supplemental Table 1).

(E) Immunohistochemistry validation of the human kidney expression and MD cell specificity of top genes that are highly expressed in the mouse MD transcriptome and suggest MD neuronal differentiation, including Semaphorin 4D (SEMA4D), Anoctamin (ANO1), Death-associated protein kinase (DAPK1), LDL-related peptide 1B (LRP1B), Synaptotagmin 8 (SYT8), Heat shock protein 90 alpha family class B member 1 (HSP90AB1). Data from the Human Protein Atlas.

(F) Tissue 3D volume projection images from optically cleared whole-mount MD-GFP kidneys immunolabeled for endogenous MD-specific GFP expression (green). Left: Tyrosin-hydroxylase (TH) co-labeling (magenta) identifies sympathetic nerve terminals. Magnified area as shown illustrates the close anatomical contact between sympathetic nerve endings and the tip of MD cell basal processes. Center: Co-labeling for TH (magenta) and calcitonin gene-related peptide (CGRP, cyan) that illuminates renal sensory nerves. Overlay and individual TH and CGRP channels are shown separately. Right: Co-labeling for synaptophysin (SYP, red) illuminates the MD (arrows)

and the adjacent renal nerve endings (intense red areas). Overlay and individual SYP and MD-GFP channels are shown separately. Bars: 50 μm.

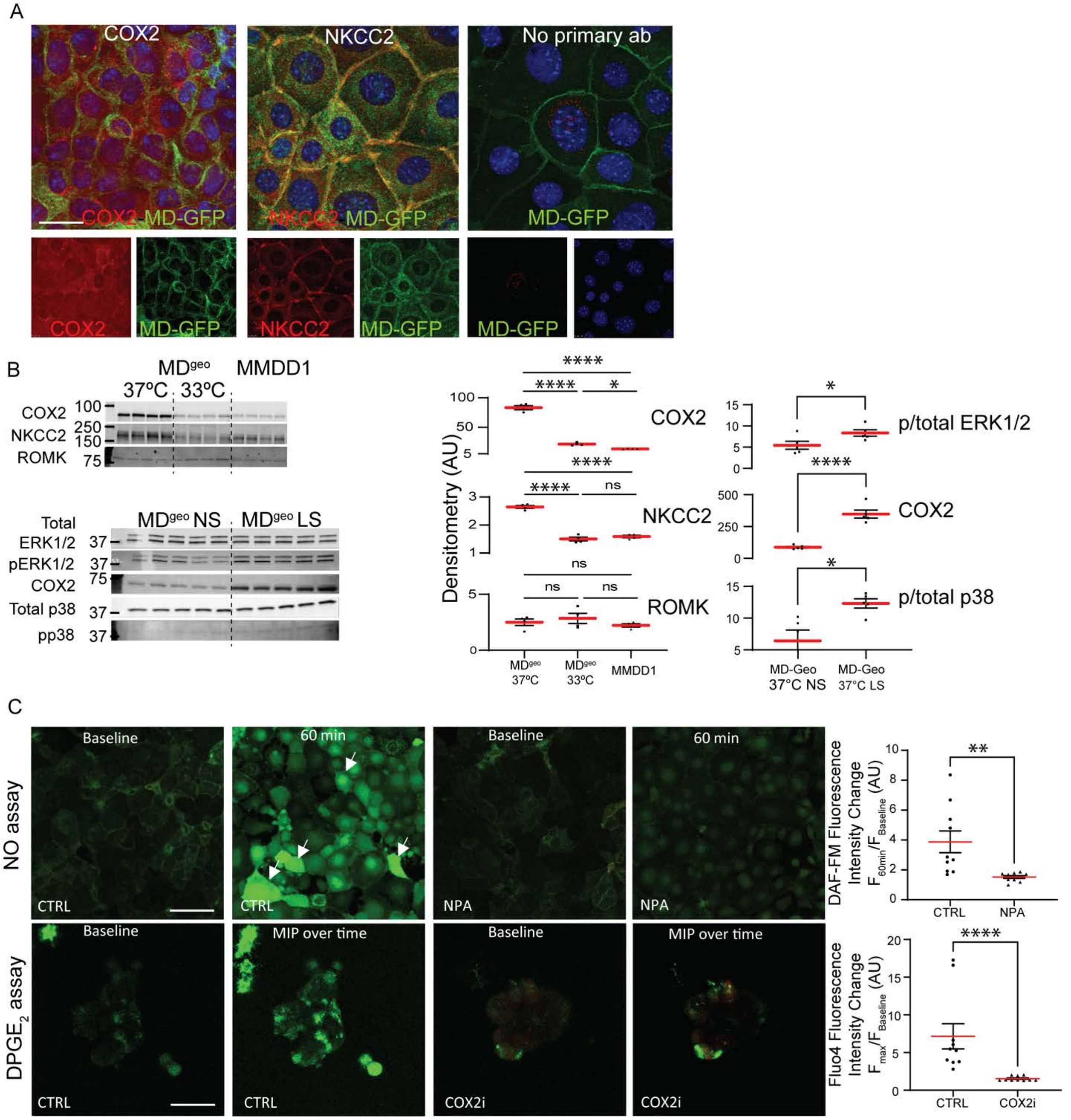

Supplemental Figure 4. Validation and characterization of the new MD cell line MD^{geo}.

(A) Immunofluorescence labeling of Cox2 and Nkcc2 in MD^{geo} cells showing cytosolic or membrane-targeted localization, respectively. Negative control shows endogenous genetic mGFP expression only. Nuclei are labeled blue with DAPI. Note the co-localization of mGFP and Nkcc2

signals (yellow in overlay) in the cell membrane. In addition to the overlay, separate red and green channels are shown at the bottom. Bar: 20 µm

(B) Immunoblots and statistical summaries of COX2, NKCC2, and ROMK expression in mMD^{geo} cells cultured at 37° and 33°C, and in the previously established but now extinct MMDD1 cell line. The effect of low salt (LS) versus normal salt (NS) culture condition on the ratio of phospho/total ERK1/2, COX2, and phospho/total p38 in differentiated (37° C) mMD^{geo} cells, n=4-5. Note the high expression of classic MD cell markers including COX2, NKCC2, and ROMK and the almost 10-fold higher expression of COX2 and NKCC2 in differentiated mMD^{geo} cells compared to either undifferentiated mMD^{geo} cells grown at 33°C or MMDD1 cells.

(C) Measurement of nitric oxide (NO, top row) and PGE_2 (bottom row) synthesis and release in MD^{geo} cells in timed control (at baseline and 60 min) and after preincubation with either selective Nos1 inhibitor NO-propyl-L-arginine (NPA, 300 mM) or selective COX-2 inhibitor SC58236 (100 nM) for Nos1-mediated NO and COX2-mediated PGE2 detection, respectively. For NO measurement, cells were loaded with the fluorescent NO indicator DAF-FM at 37 °C, and changes in DAF-FM fluorescence intensity (green) were monitored using time-lapse confocal microscopy. Maximum change in DAF-FM fluorescence intensity in the cytoplasm of MD cells was evaluated after 60 minutes and compared to baseline. Note the high DAF-FM fluorescence intensity (heterogenous pattern) in control conditions indicating NO synthesis in MD^{geo} cells. Summary of normalized DAF-FM fluorescence intensity in control condition and after NPA treatment (right panel, n=10). For the detection of MD^{geo} cell PGE_2 release, representative maximum projection images of the HEK/EP1 PGE_2 biosensor cell signal (cell Ca^{2+} detected by Fluo4) are shown at baseline and over time. Note the high HEK/EP1 PGE_2 biosensor cell signal in control conditions indicating PGE_2 release from MD^{geo} cells. Summary of HEK/EP1 PGE_2 biosensor cell signal

(normalized Fluo4 fluorescence) in control and after selective COX-2 inhibition (COX2i), n=10. Bars: 20 μm.

Data represent mean ± SEM, ns: not significant, $*p<0.05$, $**p<0.01$, $***p<0.001$, $****p<0.0001$, using t-test (between two groups) or ANOVA followed by Tukey's test (for multiple groups in B).

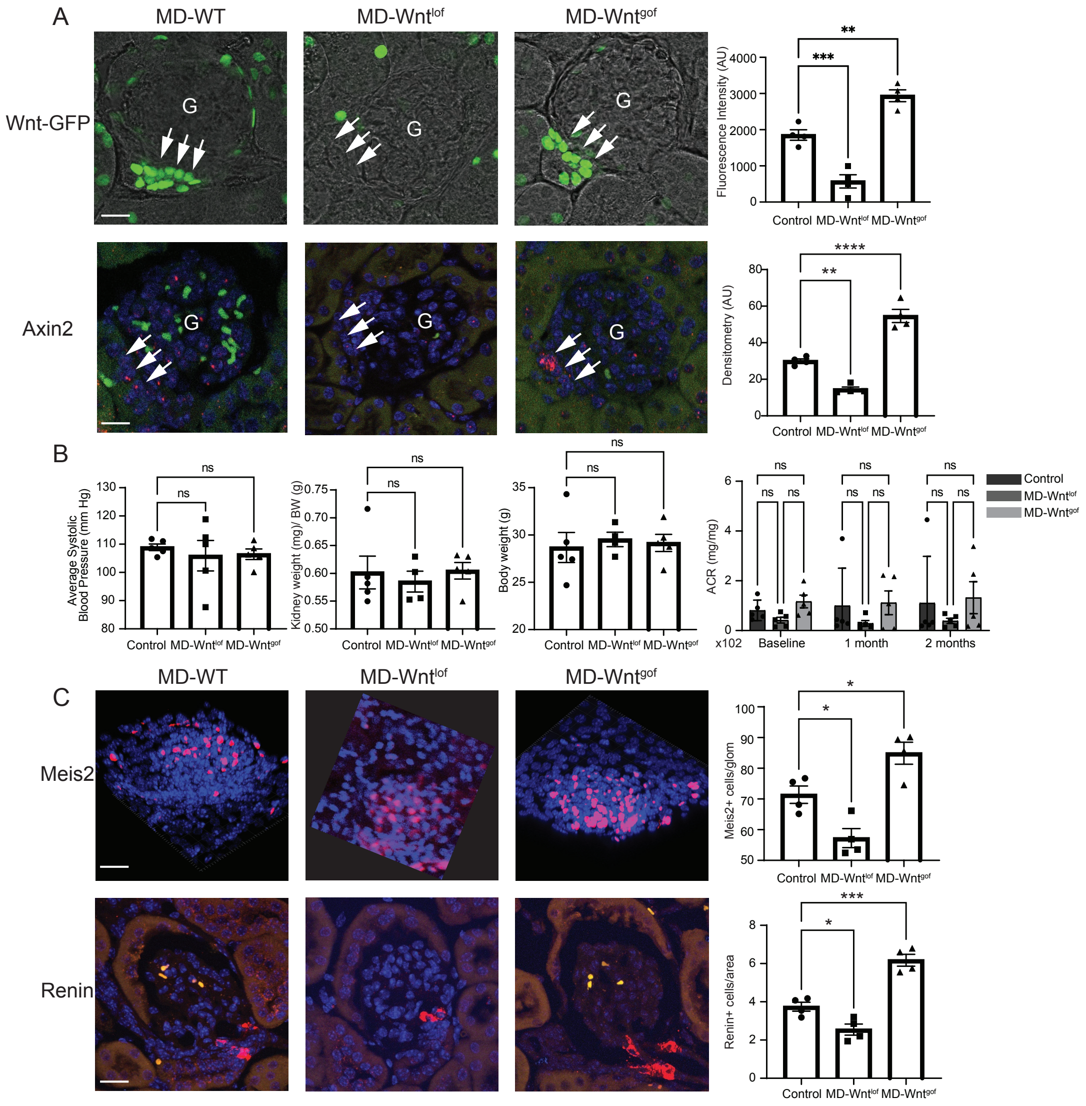

Supplemental Figure 5. Validation of altered MD Wnt activity and systemic and renal phenotypes in MD-Wnt^{gof} and MD-Wnt^{lof} mice.

(A) Fluorescence images (left) and quantification (right) of Wnt activity (top, green, from mice with nuclear TCF/Lef:H2B-GFP Wnt reporter), and Axin2 mRNA expression (bottom, red) in kidney sections of WT, MD-Wnt^{gof} and MD-Wnt^{lof} mice, n=4 (average of 5 MDs/animal). Intense labeling of MD cells in MD-Wnt^{gof} mice (arrows). G: glomerulus. Bars: 20 µm.

(B) Summary of systemic and renal parameters in the various MD-Wnt mouse models, n=4-5.

(C) Immunofluorescence images (left) and quantification (right) of Meis2^{+} (red, 3D projection images, top) and renin^{+} (red, bottom) cell number in kidney sections of WT, MD-Wnt^{gof} and MD-Wnt^{lof} mice, n=4 (average of 5 glomeruli/animal). Nuclei are labeled blue with DAPI.

Data represent mean ± SEM, ns: not significant, *p<0.05, **p<0.01, ***p<0.001, ****p<0.0001, ANOVA followed by Dunnett's test or two-way (mixed effect) ANOVA followed by Tukey's test (B, right panel).

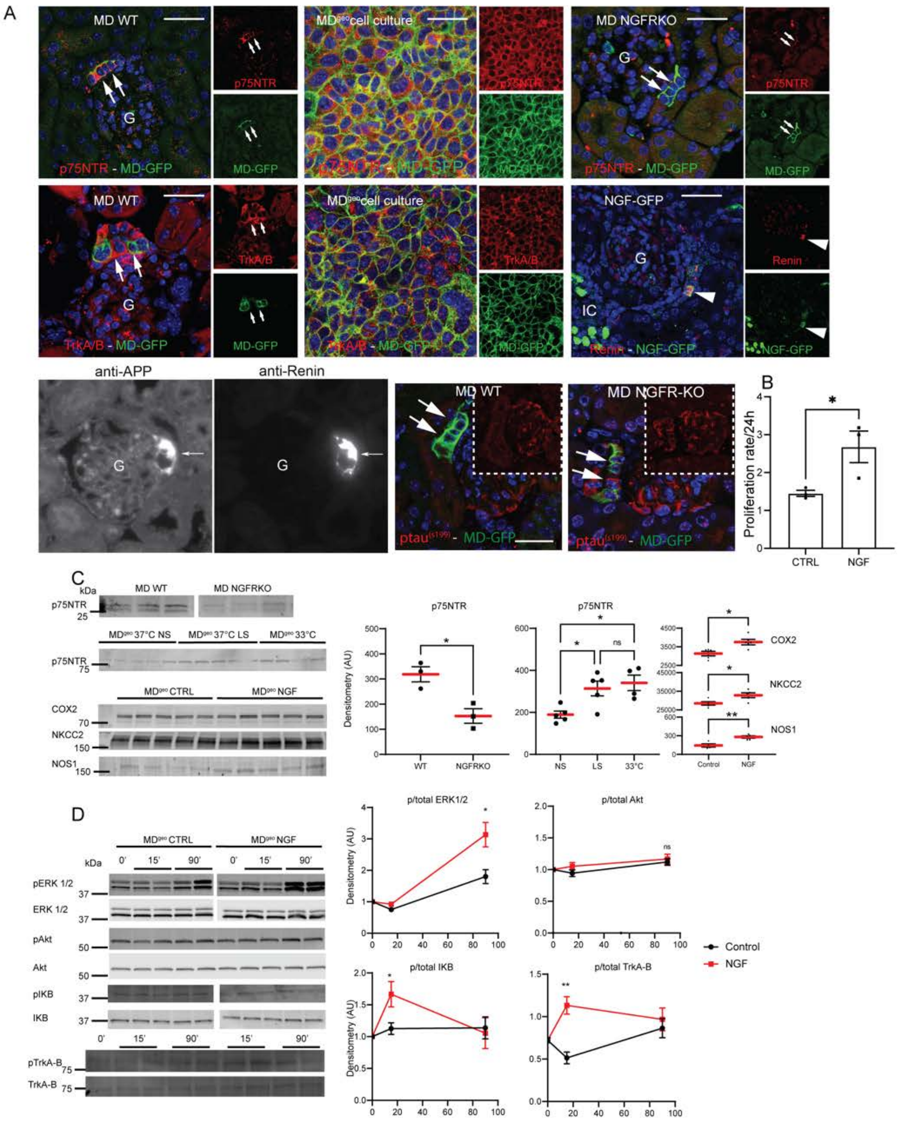

Supplemental Figure 6. Ngfr expression, signaling, and function in MD cells.

(A) p75NTR Ngfr (red, top row) immunofluorescence images (overlay, green, and red channels) showing MD cell-specific labeling (arrows, MD identified by genetic GFP expression (green)) in WT mouse kidney (left), in mMD^{geo} cells (center) and in MD-NGFR KO mouse kidney (right). Note the co-labeling of membrane-targeted GFP and Ngfr in both native MD cells in WT kidney and MD^{geo} cells (yellow in overlay) indicating Ngfr membrane localization, but the lack of Ngfr labeling in MD-NGFR KO. TrkA/B NGF receptor immunofluorescence in MD cells (arrows, center row) in WT mouse kidney (left), in mMD^{geo} cells (center) and localization of NGF

expression in renin-producing JG cells (arrowhead) in the kidney of NGF-GFP reporter mice (right). Native GFP fluorescence is shown in NGF-GFP reporter mouse kidney section, while renin immunofluorescence identified JG renin cells (red). Note the additional strong NGF-GFP signal in intercalated cells (IC) of the renal collecting duct. G: glomerulus. Cell nuclei are labeled with DAPI (blue). Bars: 50 μm. Bottom row: APP and renin immunofluorescence double-labeling in renin-expressing JG cells (arrow) in a mouse kidney section (left). p-Tau immunofluorescence (red) in MD cells (arrows) in WT and MD-NGFR KO mice. Overlay with endogenous MD-GFP signal (green), red channel shown separately in insets. Cell nuclei are labeled blue with DAPI.

(B) The effects of NGF treatment on the proliferation (cell number fold change in 24 hours) of cultured mMD^{geo} cells, n=3.

(C) Immunoblots and statistical summary of p75NTR Ngfr receptor expression in WT and MD-NGFR KO mouse kidney cortex, and in normal salt (NS) and low-salt (LS) treated mMD^{geo} cells cultured at 37°C or 33°C, and Cox2, Nkcc2, and Nos1 expression in control and NGF-treated mMD^{geo} cells at 37°C using whole cell lysates, n=3-5.

(D) Time-dependent effect of NGF treatment on MD cell MAPK (ERK1/2), Akt, NFkB and TrkA signaling using mMD^{geo} cells and immunoblotting. Baseline (0'), 15 (15') and 90 minute (90') duplicates are shown for phospho (p) or total ERK1/2, Akt, IKB, and TrkA. Statistical summaries show p/total ratio for ERK1/2, Akt, IKB, and TrkA, n=4.

Data represent mean ± SEM, ns: not significant, *p<0.05, **p<0.01, using t-test (between two groups), or ANOVA followed by Tukey's test (for multiple groups in C), or two-way ANOVA (D).

Supplemental Movies

Supplemental Movie 1. Z-stacks of the same Ng2-tdTomato (red) and Cdh5-Confetti (multicolor) kidney tissue areas visualized by serial intravital MPM imaging of the same glomeruli/kidney over several days-weeks at the indicated time points. The same preparations are shown as in Figure 1.

Supplemental Movie 2. Time-lapse imaging of MD cell calcium signaling in vivo in comparative (cc4DP, left), multi-cell (mc4DP, center), and single-cell (sc4DP, right) modes in Sox-2-GT (left and center) and MD-GT mice (right). Green: GCaMP5, red: tdTomato, greyscale: intravascular space labeled with iv injected albumin-Alexa 680. The same preparations are shown as in Figure 2B-C. MD: Macula densa, AA/EA: afferent/efferent arteriole, G: glomerulus.
