# Supplementary material for: Neuronally differentiated macula densa cells regulate tissue remodeling and regeneration in the kidney
Source: J Clin Invest. 2024 Apr 10;134(11):e174558. doi: 10.1172/JCI174558 (PMC11142747; doi:10.1172/JCI174558)

Full unedited gels for Supplemental Figure 4C

COX2

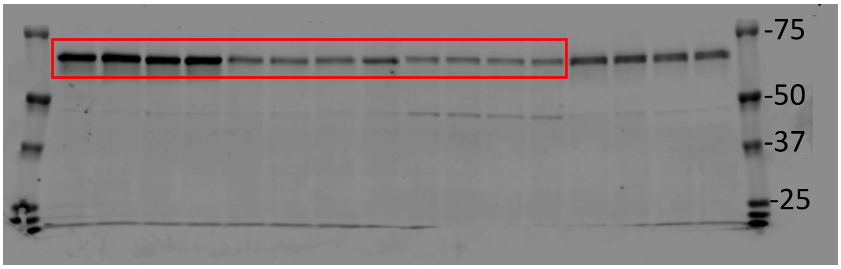

NKCC2

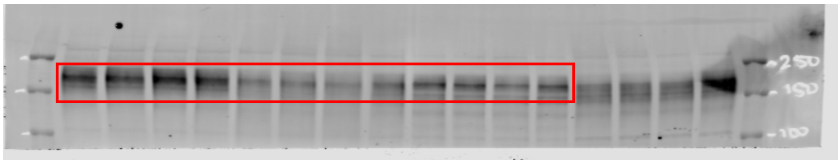

ROMK

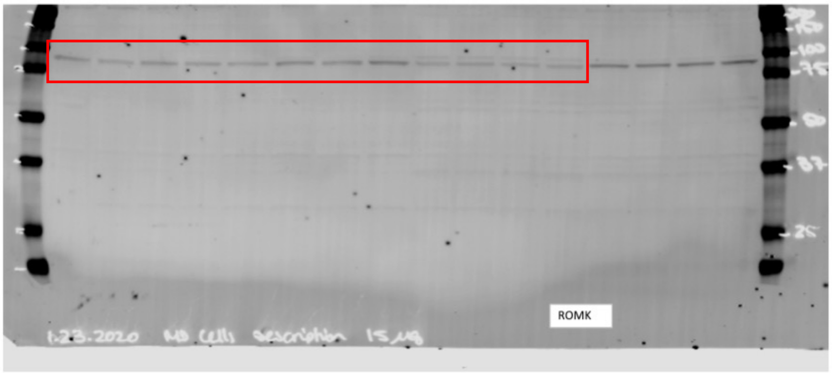

Total ERK 1/2

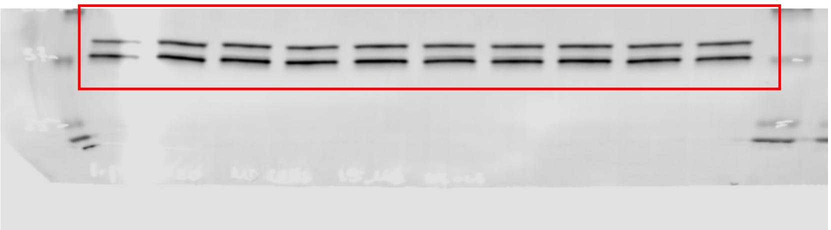

Phospho ERK 1/2

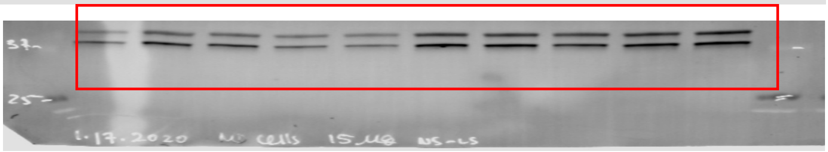

COX2

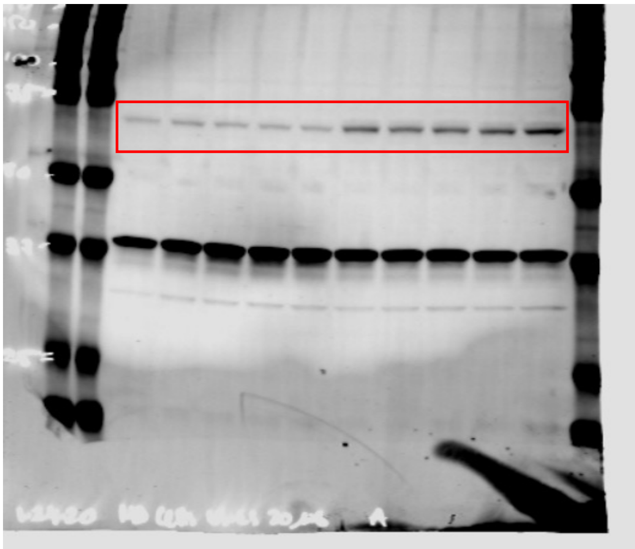

Phospho p38

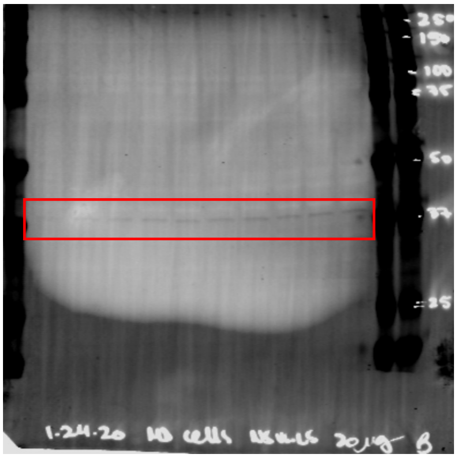

Total p38

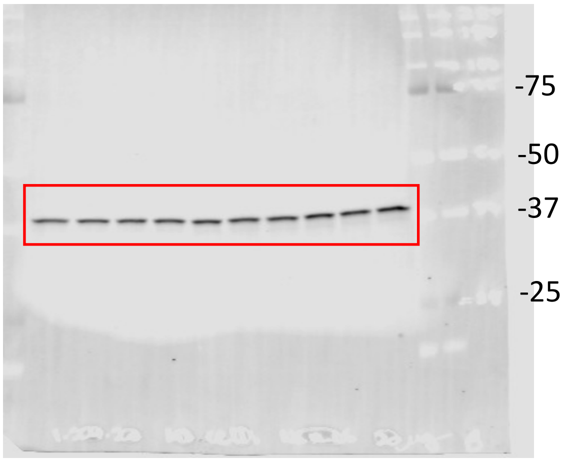

Full unedited gels for Figure 5D

CCN1

75-
50-
37-
25-

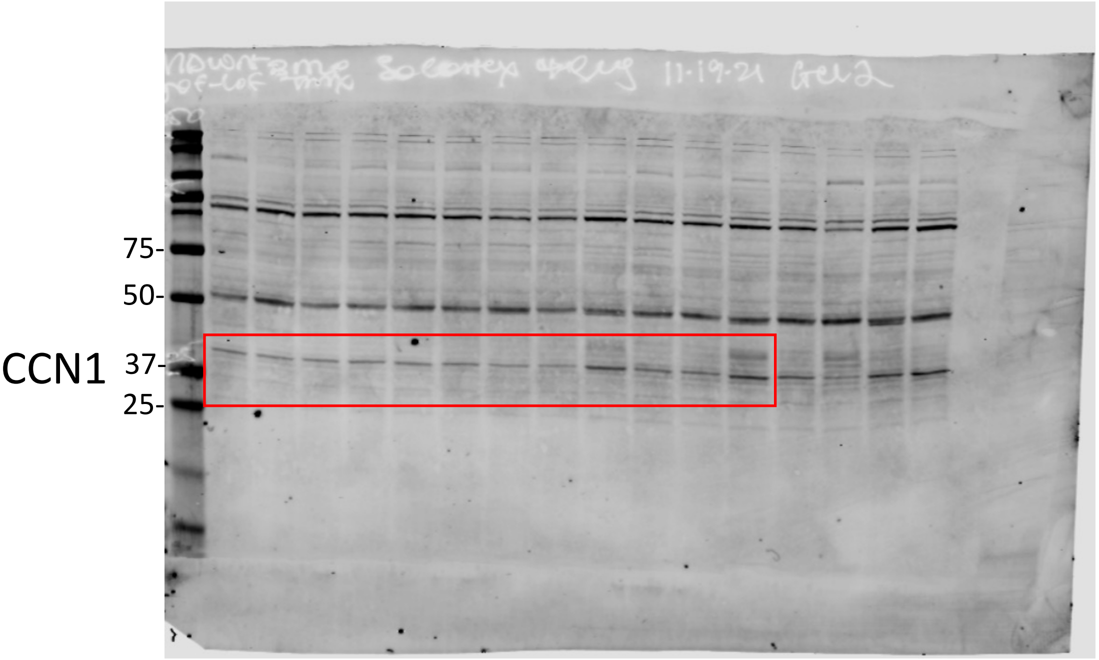

SEMA3C

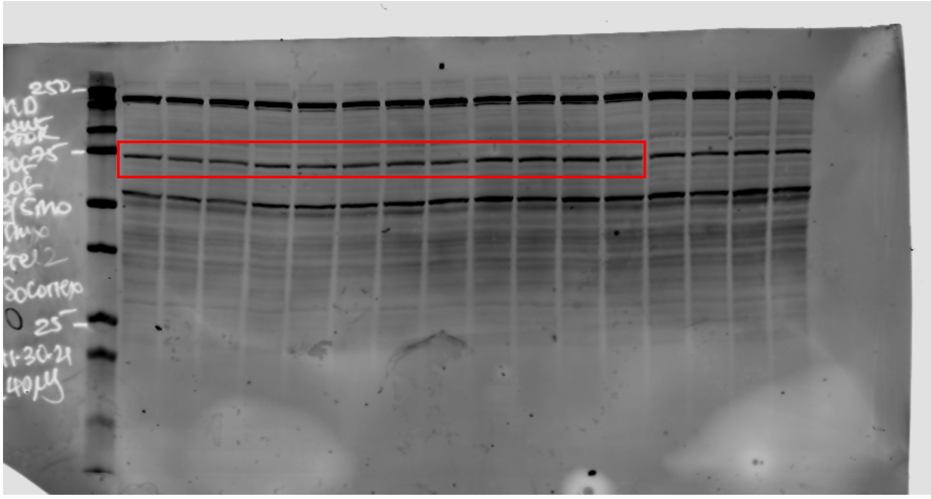

Full unedited gels for Figure 6F

CCN1

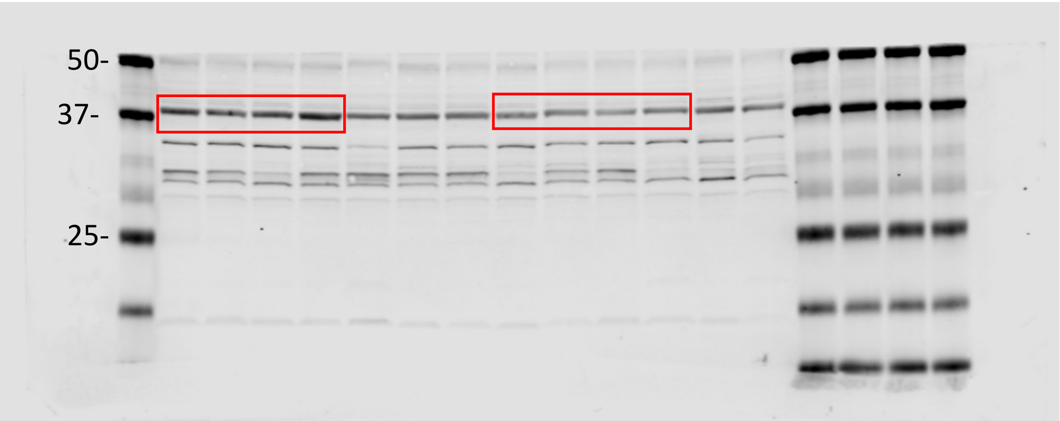

CCN3

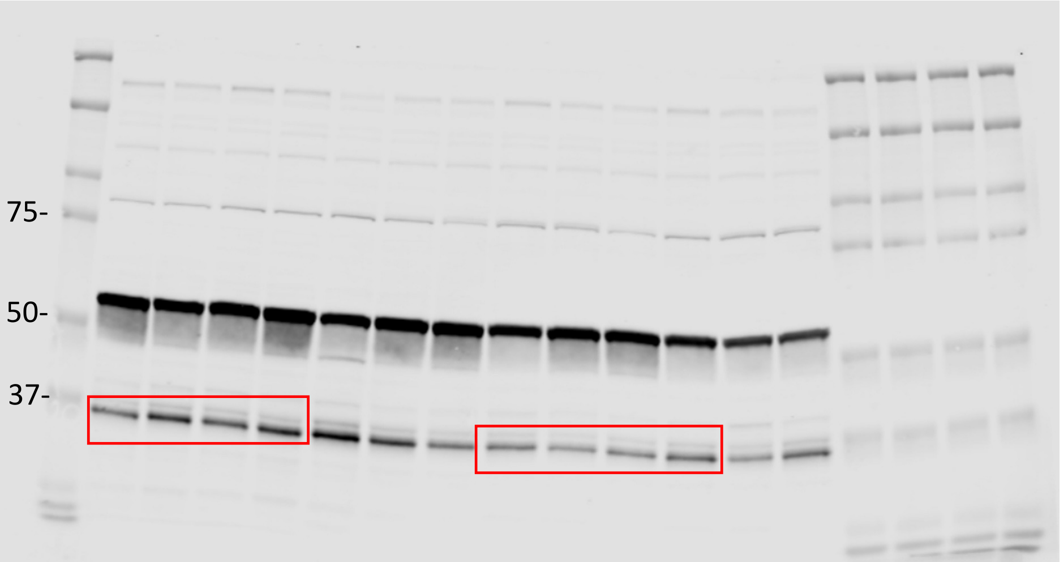

CXCL14

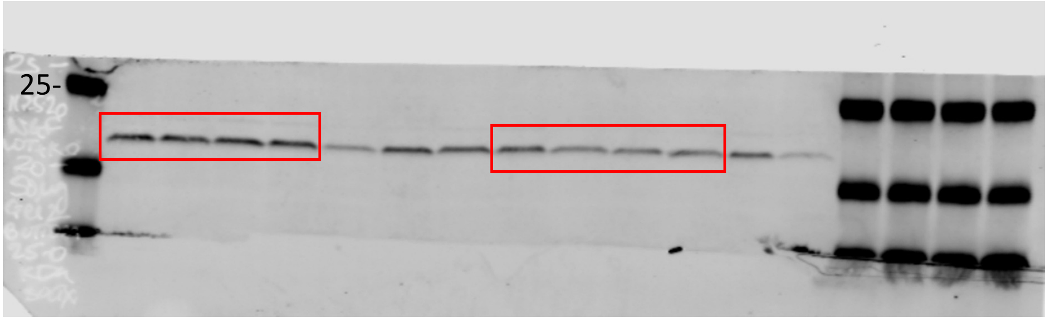

Full unedited gels for Supplemental Figure 6C

NGFR

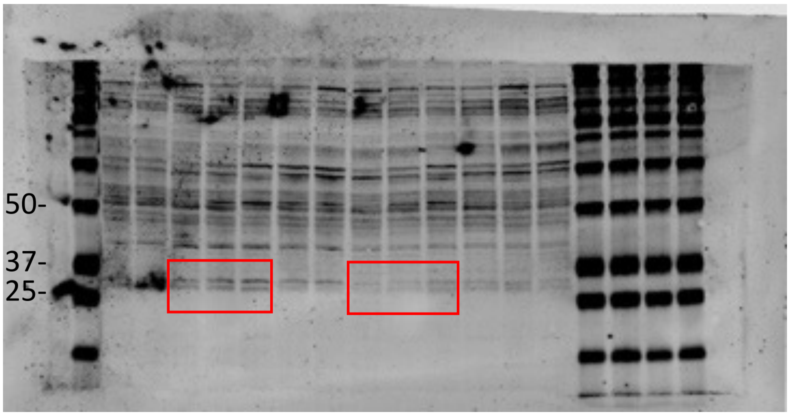

NGFR

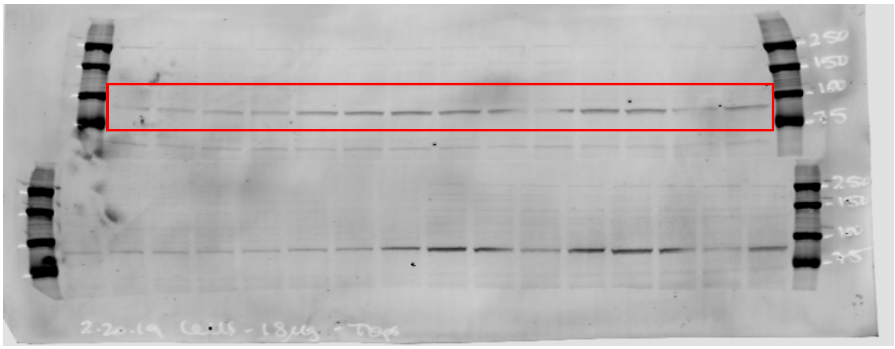

COX2

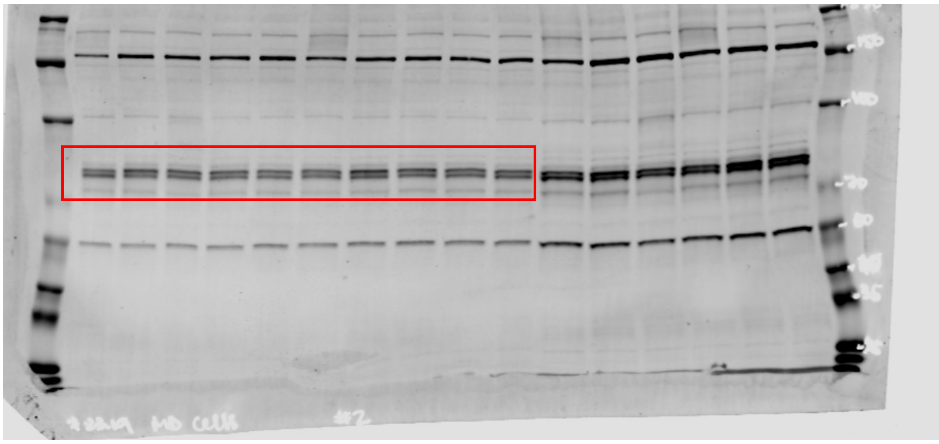

NKCC2

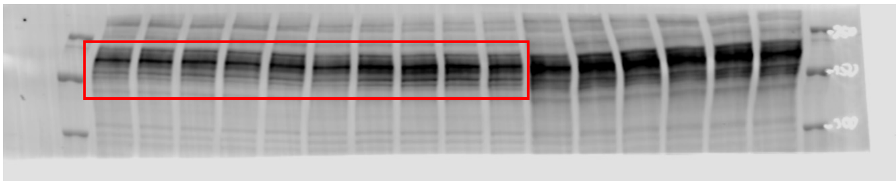

NOS1

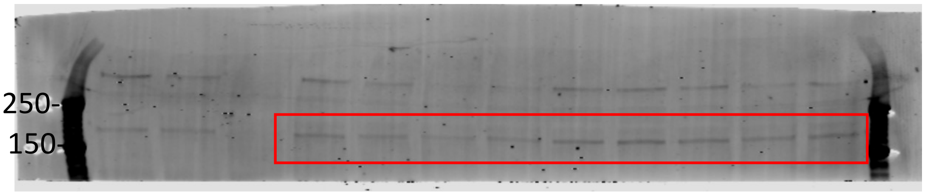

Full unedited gels for Supplemental Figure 6D

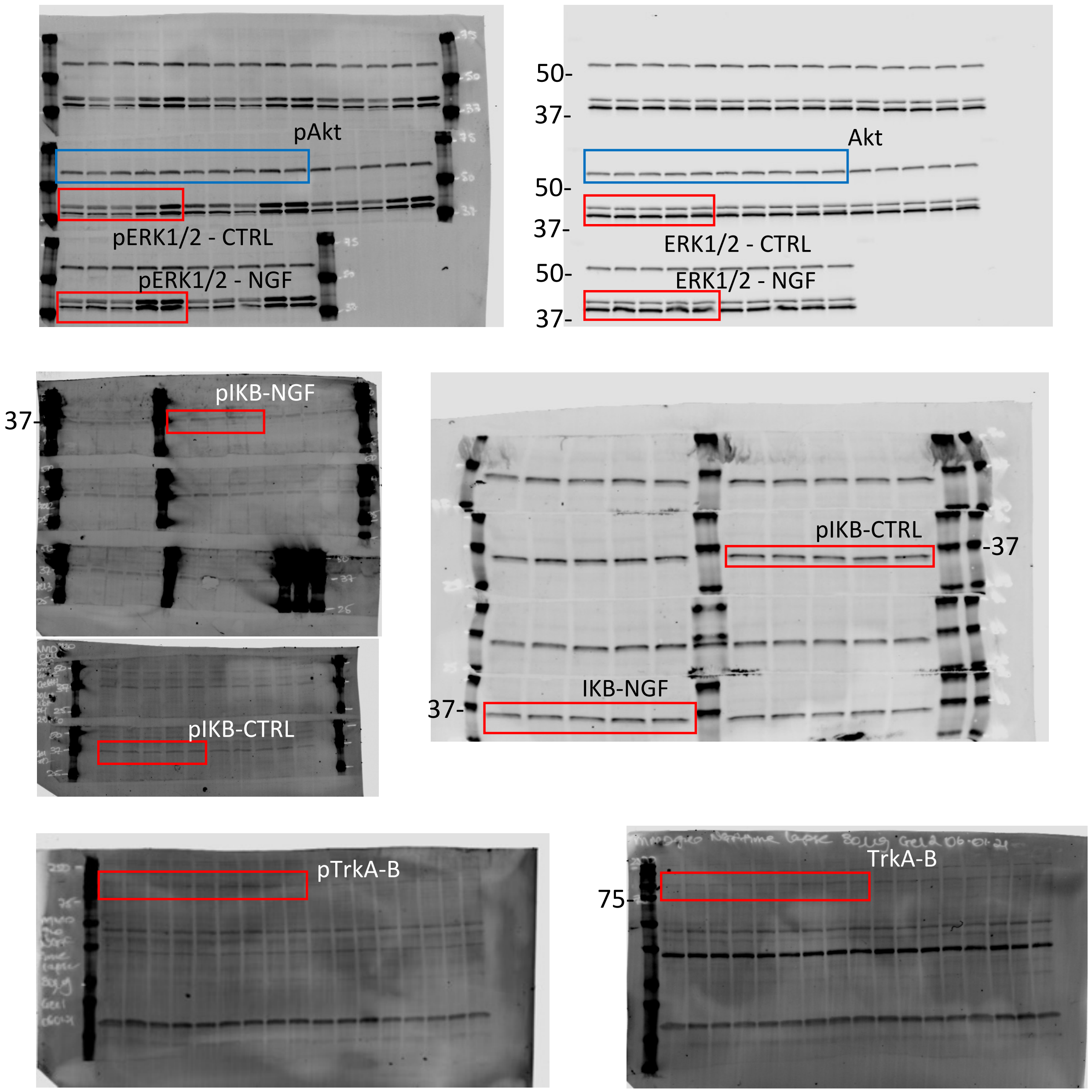

Supplement: Unedited blot and gel images [file jci-134-174558-s185.pdf]
